# Supplementary figures and images for: Automated composition of Galician Xota—tuning RNN-based composers for specific musical styles using deep Q-learning (part 2 of 2)
Source: PeerJ Comput Sci. 2023 May 15;9:e1356. doi: 10.7717/peerj-cs.1356 (PMC10280473; doi:10.7717/peerj-cs.1356)

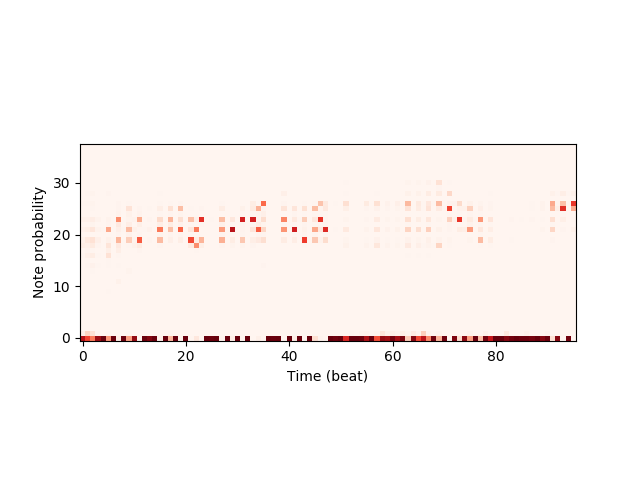

Supplement: Supplemental Information 3 [file peerj-cs-09-1356-s003.zip › Results/magenta+galician/new_rule_set/section_A/q/pre_rl96.png]

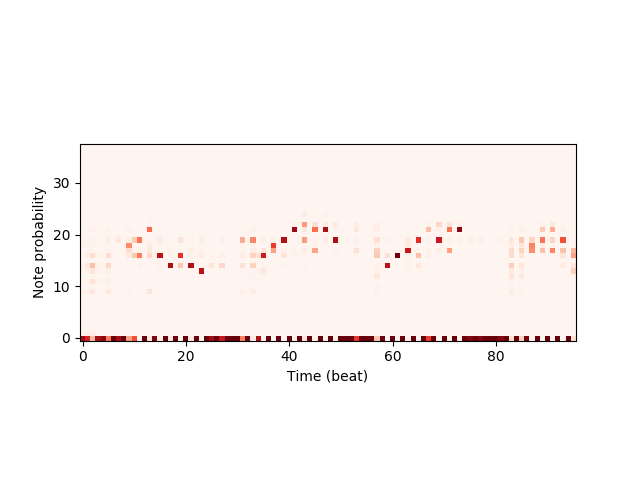

Supplement: Supplemental Information 3 [file peerj-cs-09-1356-s003.zip › Results/magenta+galician/new_rule_set/section_A/q/pre_rl70.png]

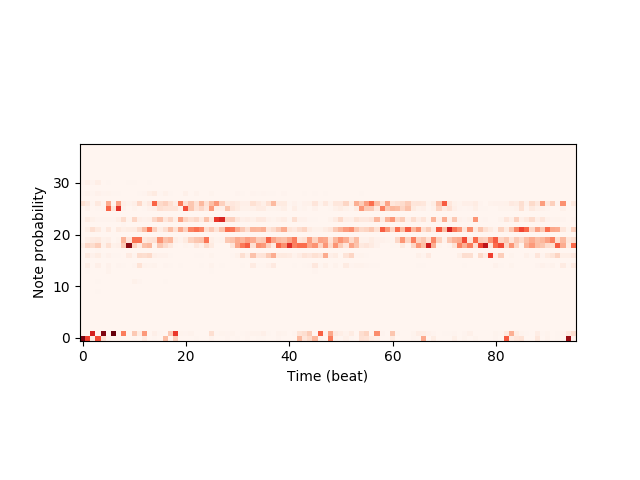

Supplement: Supplemental Information 3 [file peerj-cs-09-1356-s003.zip › Results/magenta+galician/new_rule_set/section_A/q/pre_rl83.png]

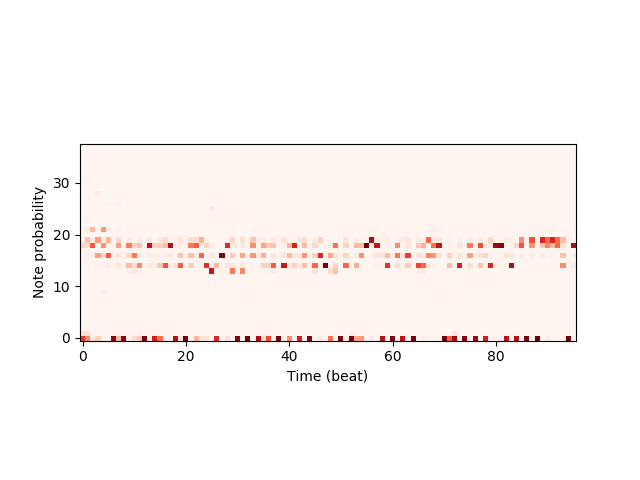

Supplement: Supplemental Information 3 [file peerj-cs-09-1356-s003.zip › Results/magenta+galician/new_rule_set/section_A/q/pre_rl90.png]

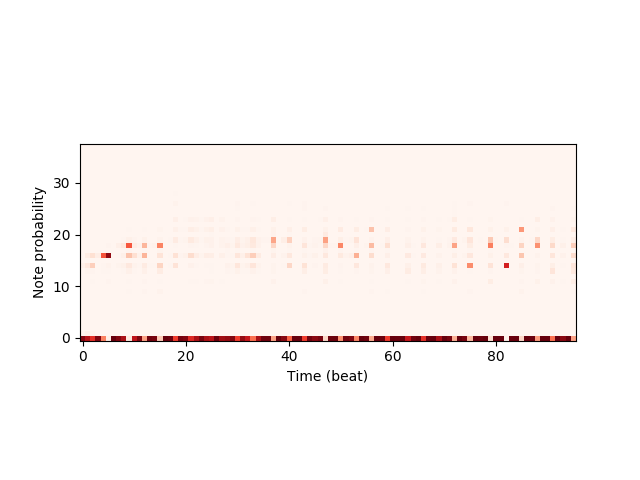

Supplement: Supplemental Information 3 [file peerj-cs-09-1356-s003.zip › Results/magenta+galician/new_rule_set/section_B/q/pre_rl10.png]

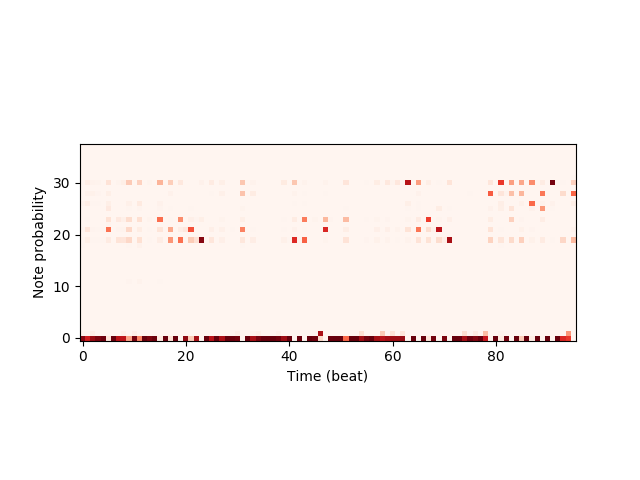

Supplement: Supplemental Information 3 [file peerj-cs-09-1356-s003.zip › Results/magenta+galician/new_rule_set/section_B/q/pre_rl58.png]

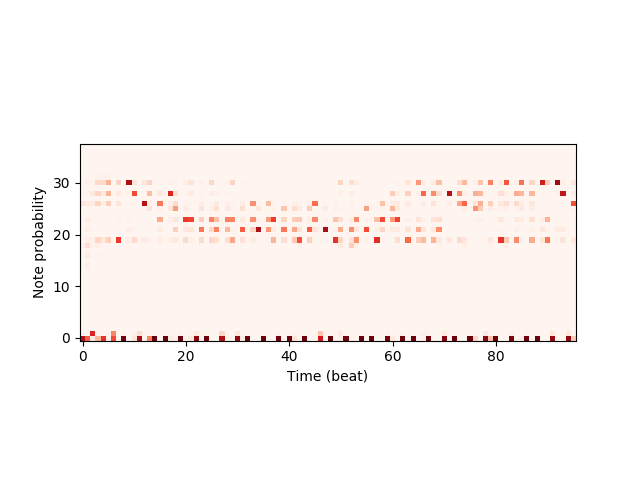

Supplement: Supplemental Information 3 [file peerj-cs-09-1356-s003.zip › Results/magenta+galician/new_rule_set/section_B/q/pre_rl31.png]

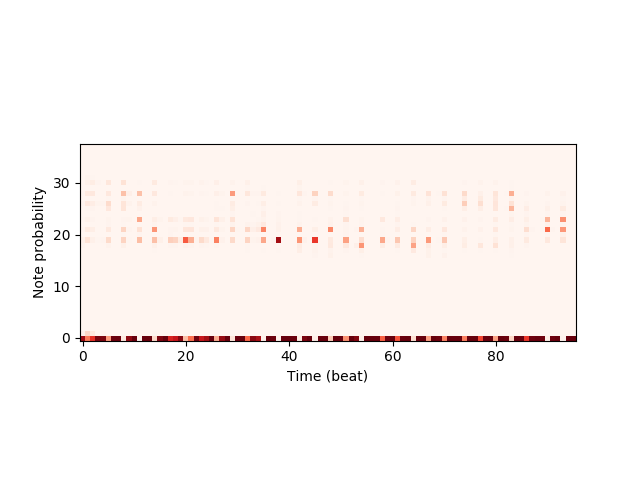

Supplement: Supplemental Information 3 [file peerj-cs-09-1356-s003.zip › Results/magenta+galician/new_rule_set/section_B/q/pre_rl42.png]

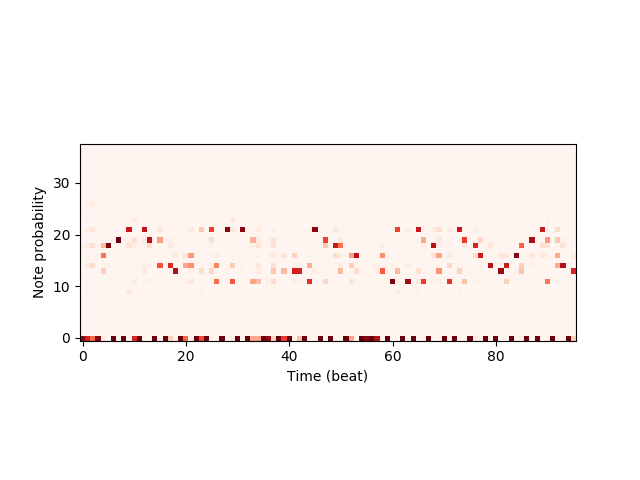

Supplement: Supplemental Information 3 [file peerj-cs-09-1356-s003.zip › Results/magenta+galician/new_rule_set/section_B/q/pre_rl34.png]

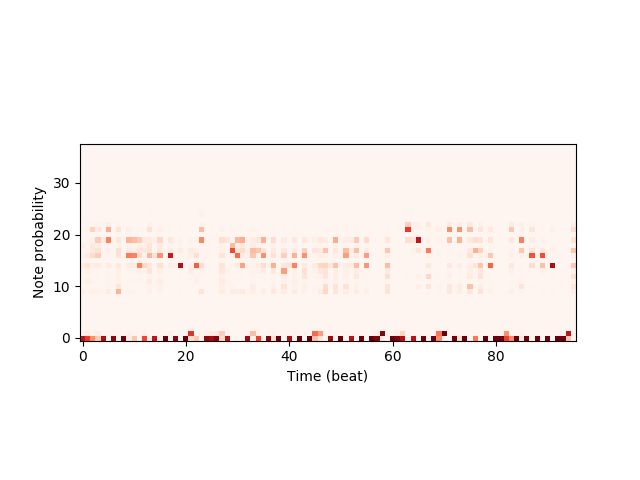

Supplement: Supplemental Information 3 [file peerj-cs-09-1356-s003.zip › Results/magenta+galician/new_rule_set/section_B/q/pre_rl35.png]

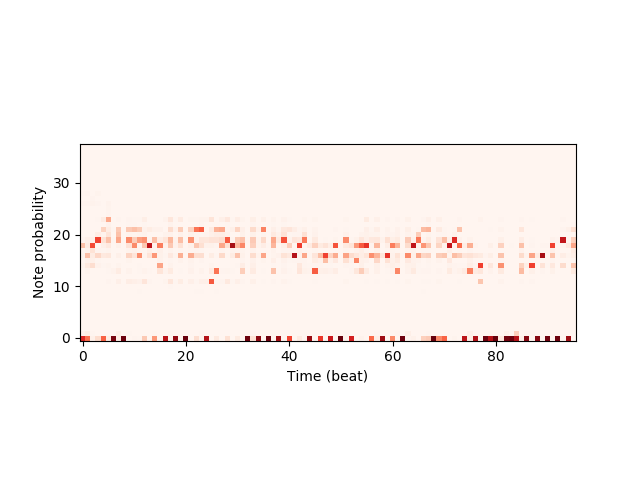

Supplement: Supplemental Information 3 [file peerj-cs-09-1356-s003.zip › Results/magenta+galician/new_rule_set/section_B/q/pre_rl36.png]

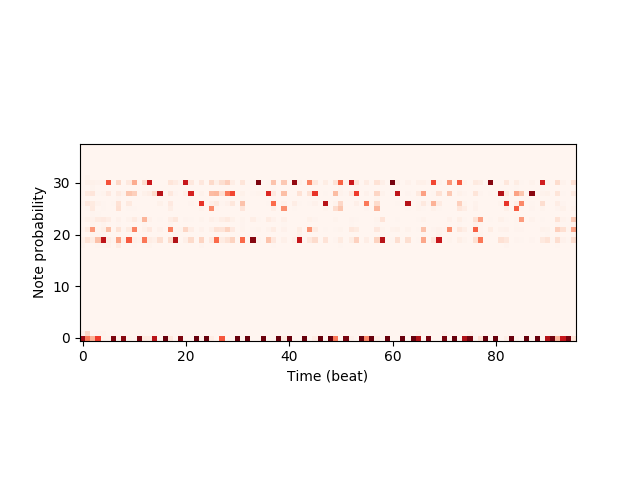

Supplement: Supplemental Information 3 [file peerj-cs-09-1356-s003.zip › Results/magenta+galician/new_rule_set/section_B/q/pre_rl56.png]

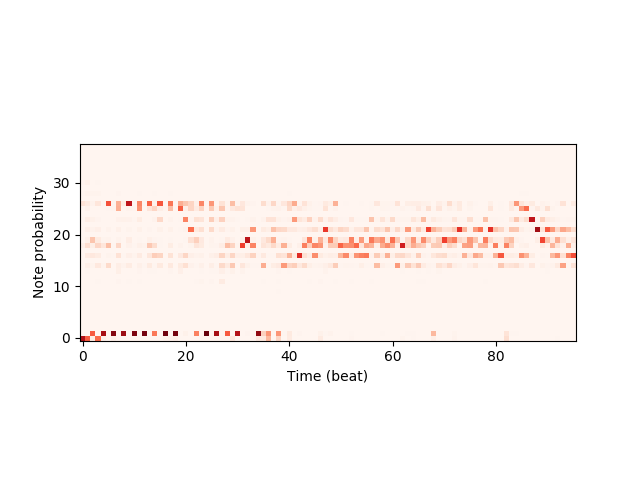

Supplement: Supplemental Information 3 [file peerj-cs-09-1356-s003.zip › Results/magenta+galician/new_rule_set/section_B/q/pre_rl11.png]

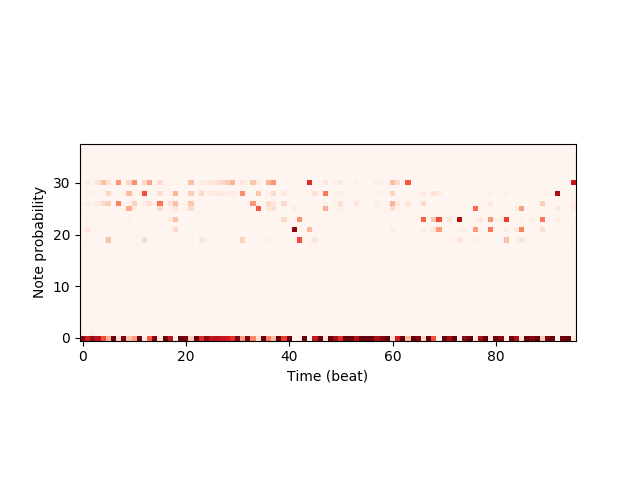

Supplement: Supplemental Information 3 [file peerj-cs-09-1356-s003.zip › Results/magenta+galician/new_rule_set/section_B/q/pre_rl7.png]

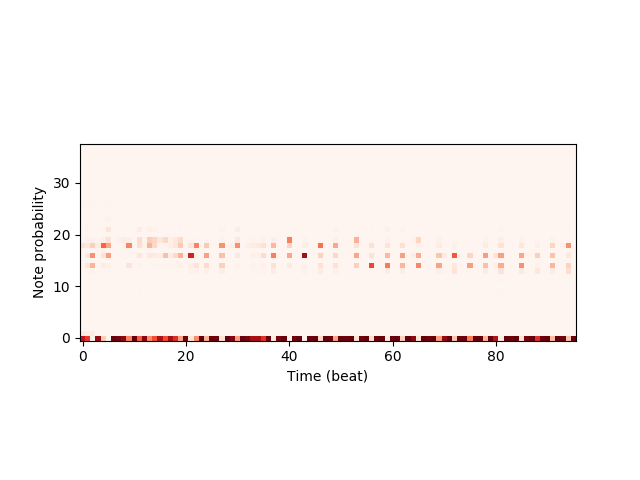

Supplement: Supplemental Information 3 [file peerj-cs-09-1356-s003.zip › Results/magenta+galician/new_rule_set/section_B/q/pre_rl20.png]

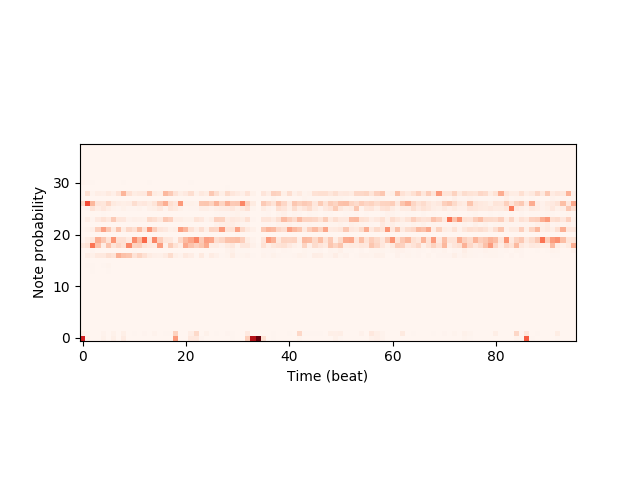

Supplement: Supplemental Information 3 [file peerj-cs-09-1356-s003.zip › Results/magenta+galician/new_rule_set/section_B/q/pre_rl37.png]

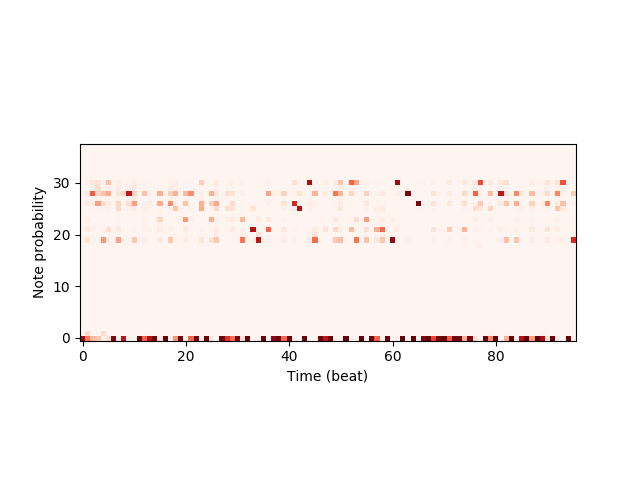

Supplement: Supplemental Information 3 [file peerj-cs-09-1356-s003.zip › Results/magenta+galician/new_rule_set/section_B/q/pre_rl46.png]

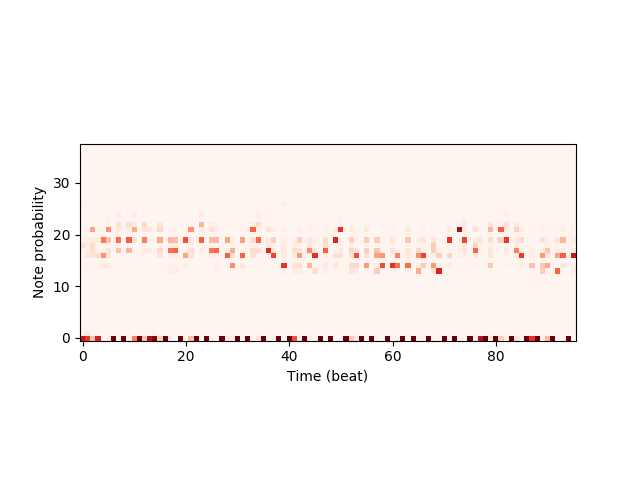

Supplement: Supplemental Information 3 [file peerj-cs-09-1356-s003.zip › Results/magenta+galician/new_rule_set/section_B/q/pre_rl54.png]

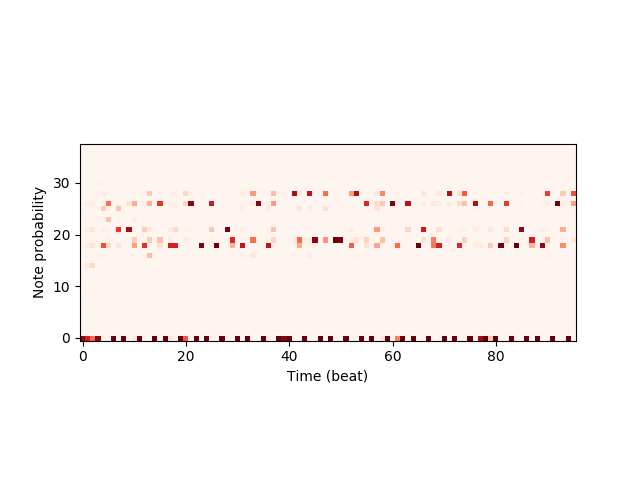

Supplement: Supplemental Information 3 [file peerj-cs-09-1356-s003.zip › Results/magenta+galician/new_rule_set/section_B/q/pre_rl68.png]

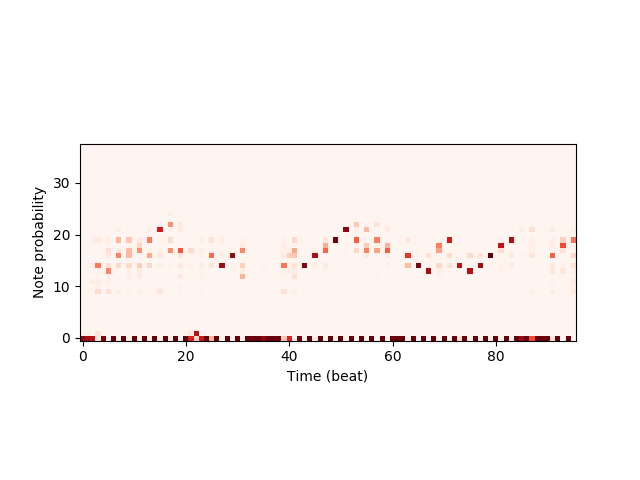

Supplement: Supplemental Information 3 [file peerj-cs-09-1356-s003.zip › Results/magenta+galician/new_rule_set/section_B/q/pre_rl71.png]

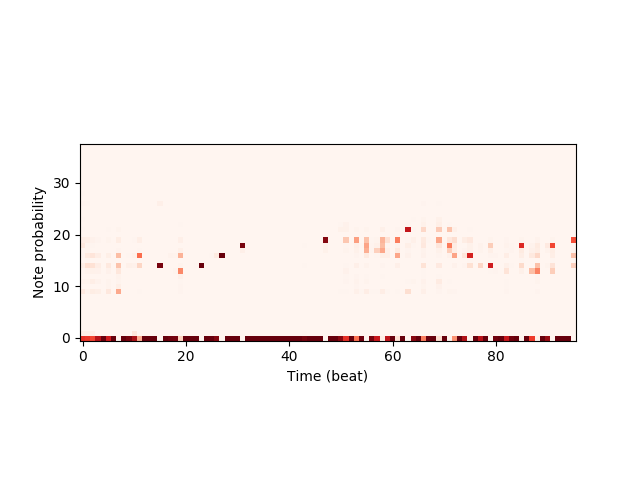

Supplement: Supplemental Information 3 [file peerj-cs-09-1356-s003.zip › Results/magenta+galician/new_rule_set/section_B/q/pre_rl73.png]

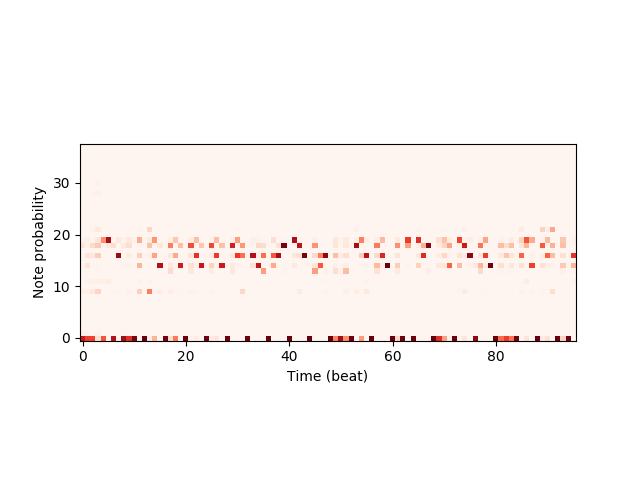

Supplement: Supplemental Information 3 [file peerj-cs-09-1356-s003.zip › Results/magenta+galician/new_rule_set/section_B/q/pre_rl87.png]

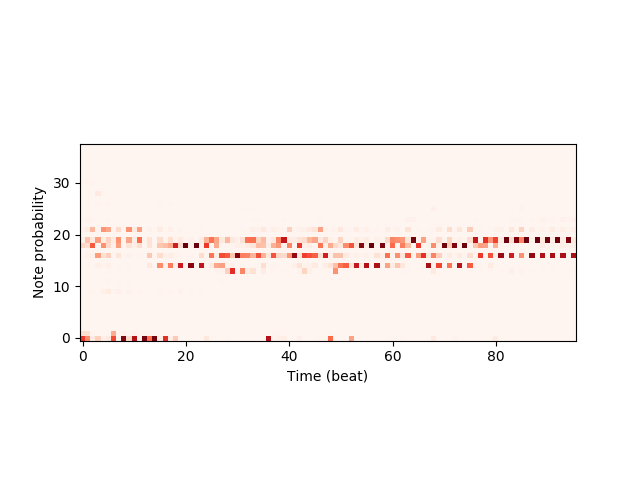

Supplement: Supplemental Information 3 [file peerj-cs-09-1356-s003.zip › Results/magenta+galician/new_rule_set/section_B/q/pre_rl97.png]

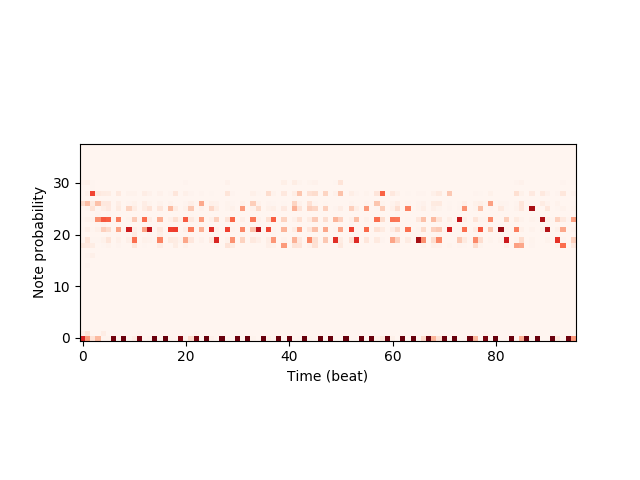

Supplement: Supplemental Information 3 [file peerj-cs-09-1356-s003.zip › Results/magenta+galician/new_rule_set/section_B/q/pre_rl15.png]

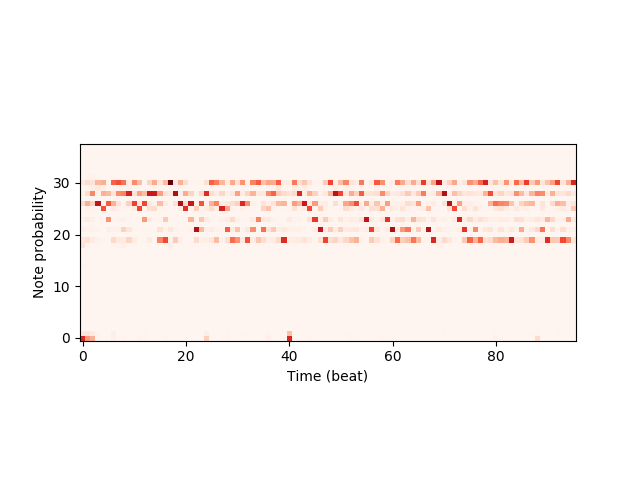

Supplement: Supplemental Information 3 [file peerj-cs-09-1356-s003.zip › Results/magenta+galician/new_rule_set/section_B/q/pre_rl41.png]

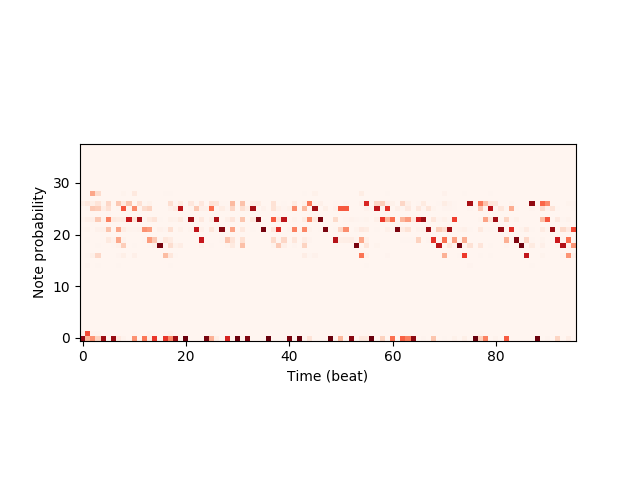

Supplement: Supplemental Information 3 [file peerj-cs-09-1356-s003.zip › Results/magenta+galician/new_rule_set/section_B/q/pre_rl43.png]

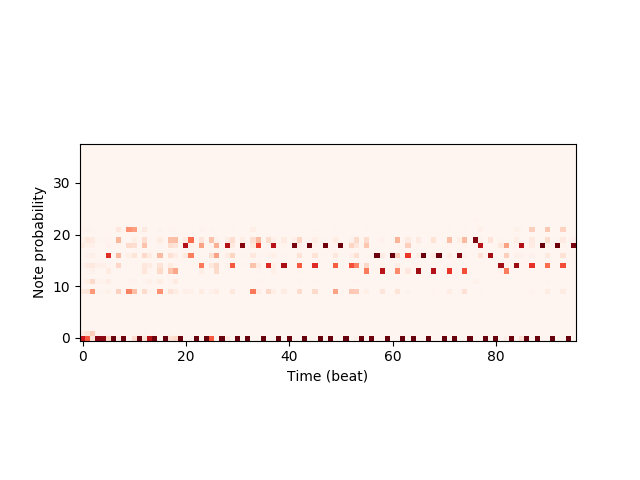

Supplement: Supplemental Information 3 [file peerj-cs-09-1356-s003.zip › Results/magenta+galician/new_rule_set/section_B/q/pre_rl63.png]

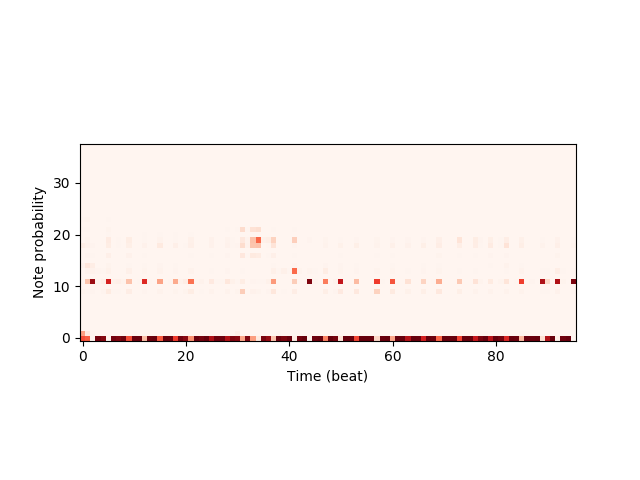

Supplement: Supplemental Information 3 [file peerj-cs-09-1356-s003.zip › Results/magenta+galician/new_rule_set/section_B/q/pre_rl80.png]

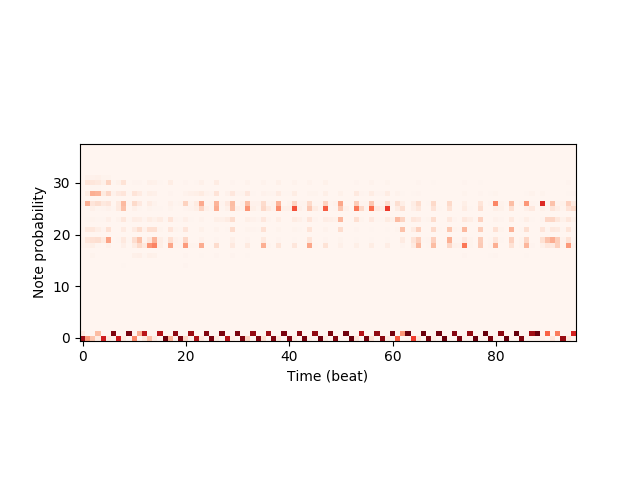

Supplement: Supplemental Information 3 [file peerj-cs-09-1356-s003.zip › Results/magenta+galician/new_rule_set/section_B/q/pre_rl12.png]

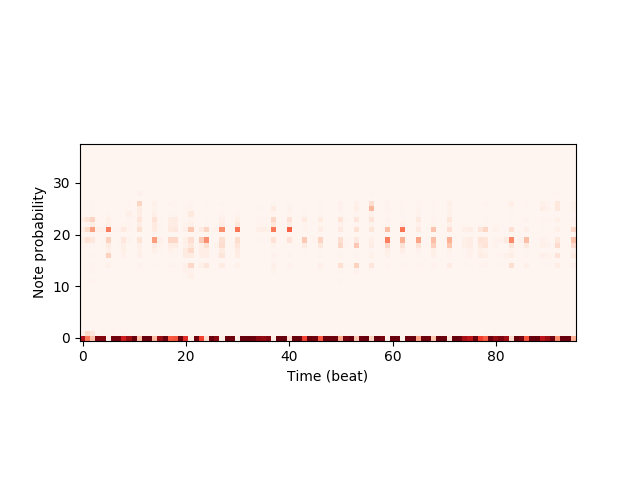

Supplement: Supplemental Information 3 [file peerj-cs-09-1356-s003.zip › Results/magenta+galician/new_rule_set/section_B/q/pre_rl2.png]

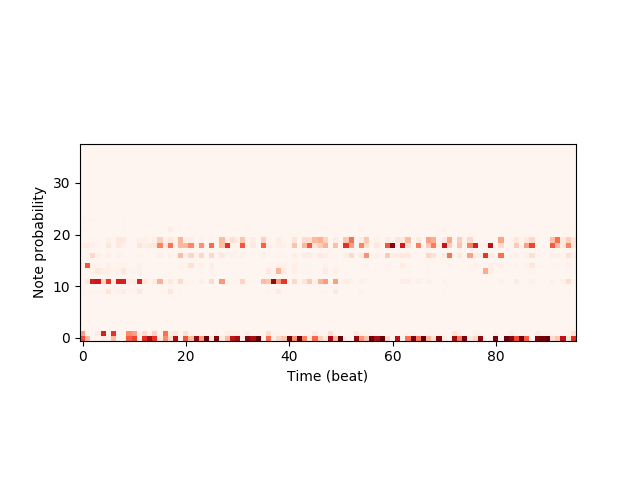

Supplement: Supplemental Information 3 [file peerj-cs-09-1356-s003.zip › Results/magenta+galician/new_rule_set/section_B/q/pre_rl8.png]

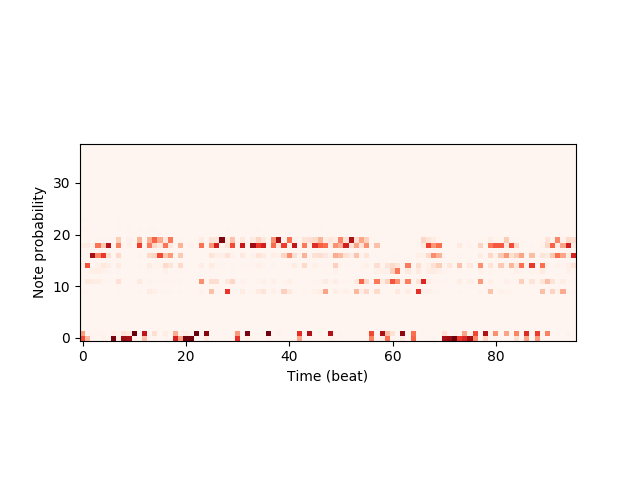

Supplement: Supplemental Information 3 [file peerj-cs-09-1356-s003.zip › Results/magenta+galician/new_rule_set/section_B/q/pre_rl25.png]

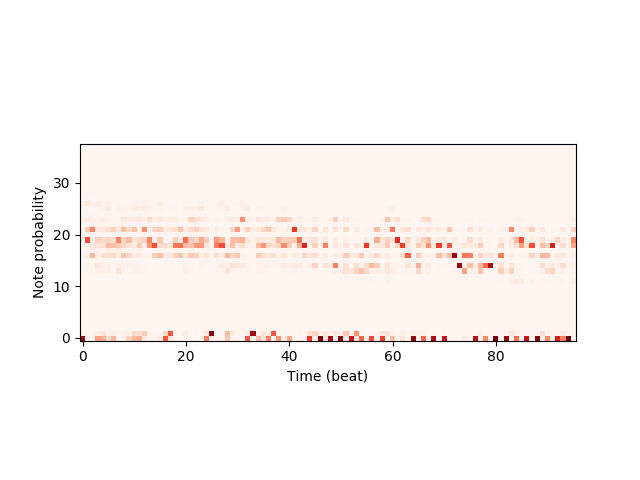

Supplement: Supplemental Information 3 [file peerj-cs-09-1356-s003.zip › Results/magenta+galician/new_rule_set/section_B/q/pre_rl69.png]

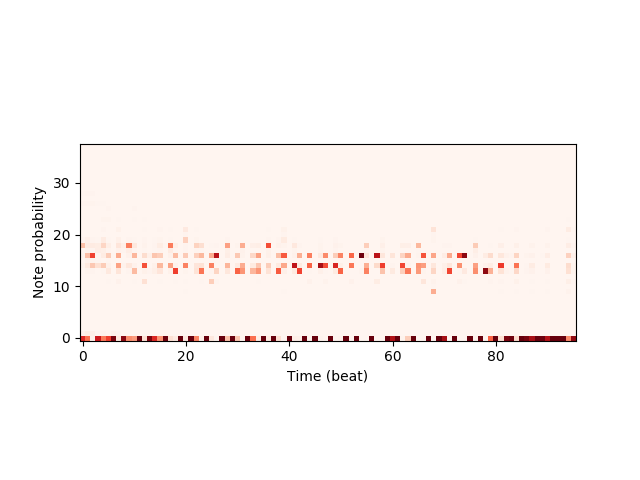

Supplement: Supplemental Information 3 [file peerj-cs-09-1356-s003.zip › Results/magenta+galician/new_rule_set/section_B/q/pre_rl82.png]

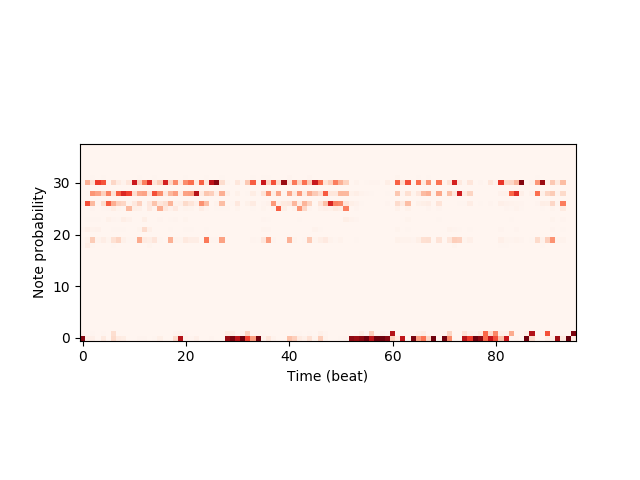

Supplement: Supplemental Information 3 [file peerj-cs-09-1356-s003.zip › Results/magenta+galician/new_rule_set/section_B/q/pre_rl89.png]

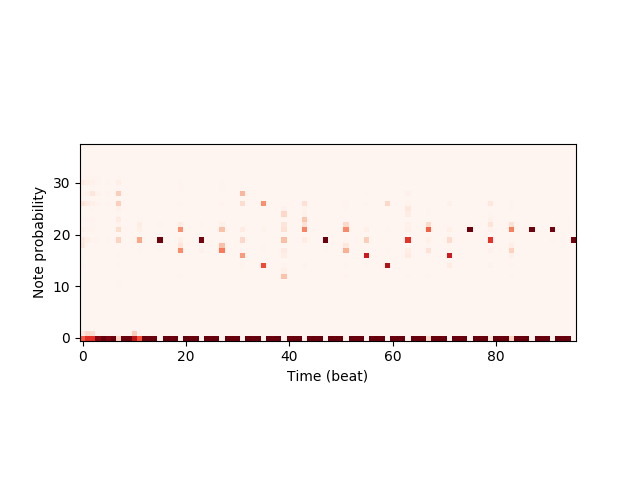

Supplement: Supplemental Information 3 [file peerj-cs-09-1356-s003.zip › Results/magenta+galician/new_rule_set/section_B/q/pre_rl22.png]

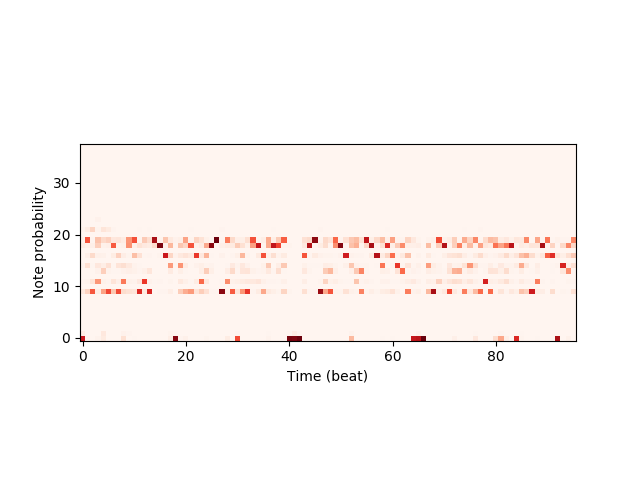

Supplement: Supplemental Information 3 [file peerj-cs-09-1356-s003.zip › Results/magenta+galician/new_rule_set/section_B/q/pre_rl29.png]

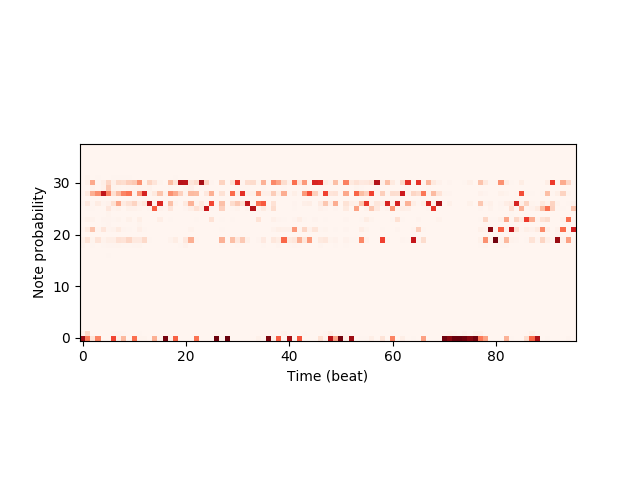

Supplement: Supplemental Information 3 [file peerj-cs-09-1356-s003.zip › Results/magenta+galician/new_rule_set/section_B/q/pre_rl51.png]

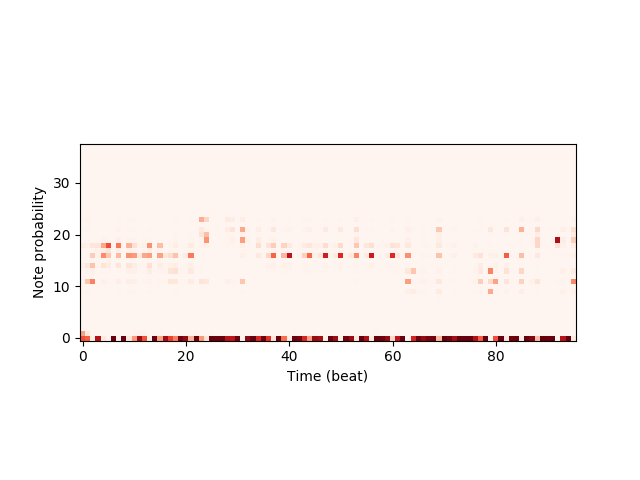

Supplement: Supplemental Information 3 [file peerj-cs-09-1356-s003.zip › Results/magenta+galician/new_rule_set/section_B/q/pre_rl57.png]

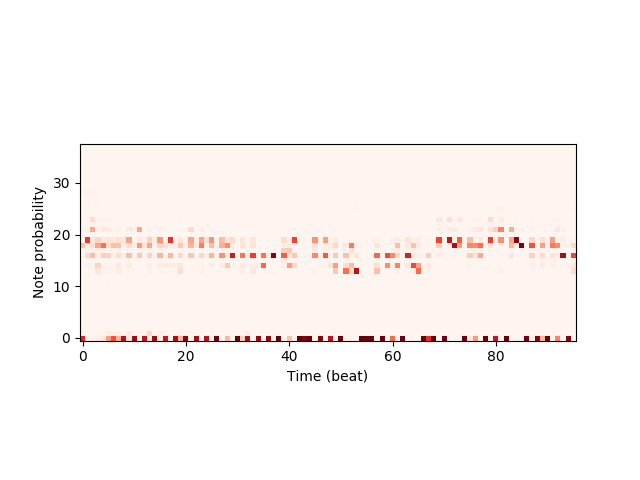

Supplement: Supplemental Information 3 [file peerj-cs-09-1356-s003.zip › Results/magenta+galician/new_rule_set/section_B/q/pre_rl72.png]

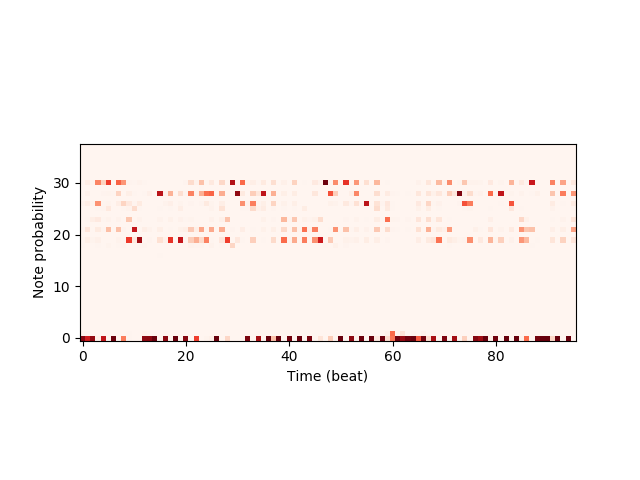

Supplement: Supplemental Information 3 [file peerj-cs-09-1356-s003.zip › Results/magenta+galician/new_rule_set/section_B/q/pre_rl94.png]

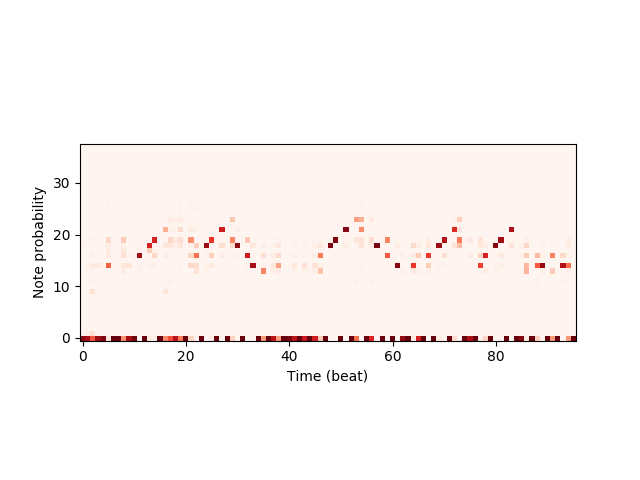

Supplement: Supplemental Information 3 [file peerj-cs-09-1356-s003.zip › Results/magenta+galician/new_rule_set/section_B/q/pre_rl44.png]

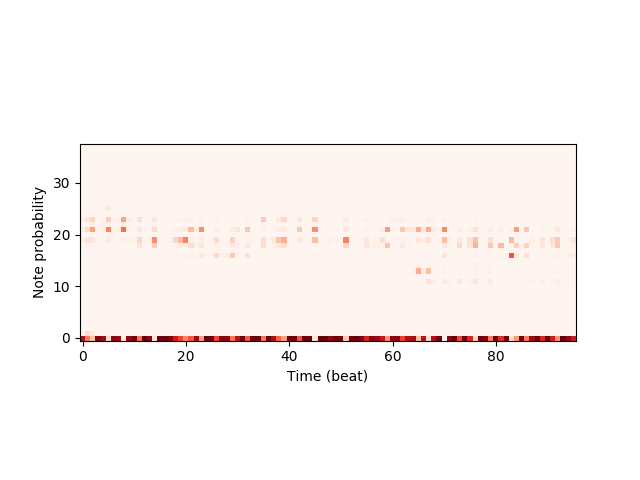

Supplement: Supplemental Information 3 [file peerj-cs-09-1356-s003.zip › Results/magenta+galician/new_rule_set/section_B/q/pre_rl45.png]

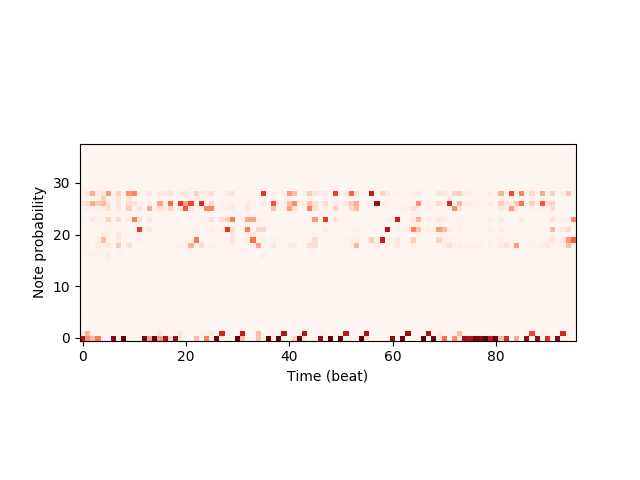

Supplement: Supplemental Information 3 [file peerj-cs-09-1356-s003.zip › Results/magenta+galician/new_rule_set/section_B/q/pre_rl33.png]

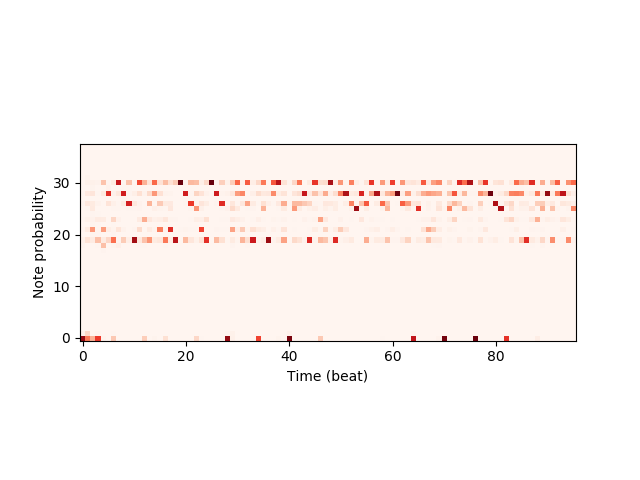

Supplement: Supplemental Information 3 [file peerj-cs-09-1356-s003.zip › Results/magenta+galician/new_rule_set/section_B/q/pre_rl38.png]

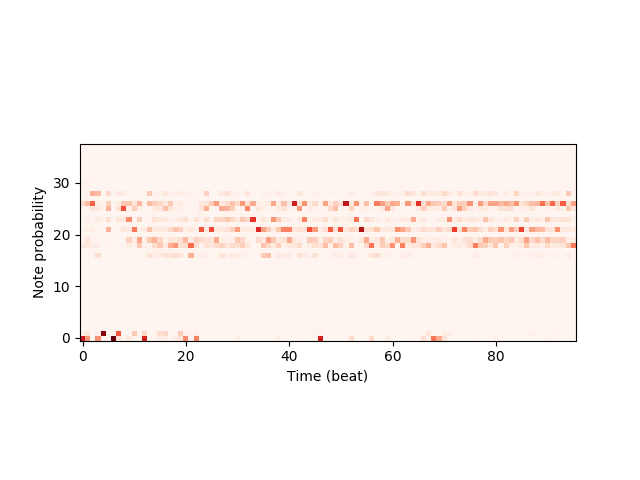

Supplement: Supplemental Information 3 [file peerj-cs-09-1356-s003.zip › Results/magenta+galician/new_rule_set/section_B/q/pre_rl4.png]

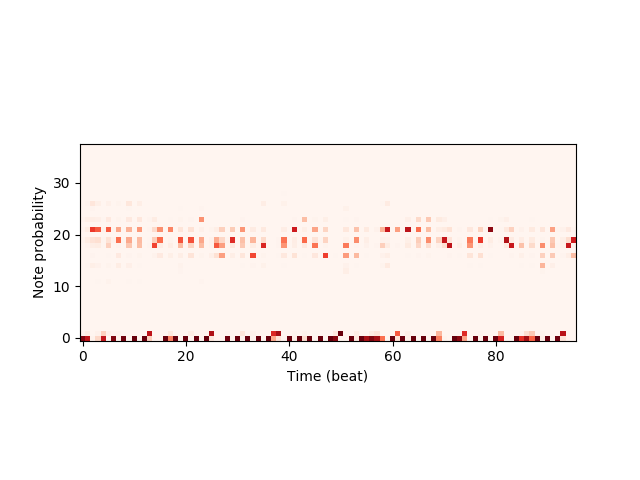

Supplement: Supplemental Information 3 [file peerj-cs-09-1356-s003.zip › Results/magenta+galician/new_rule_set/section_B/q/pre_rl48.png]

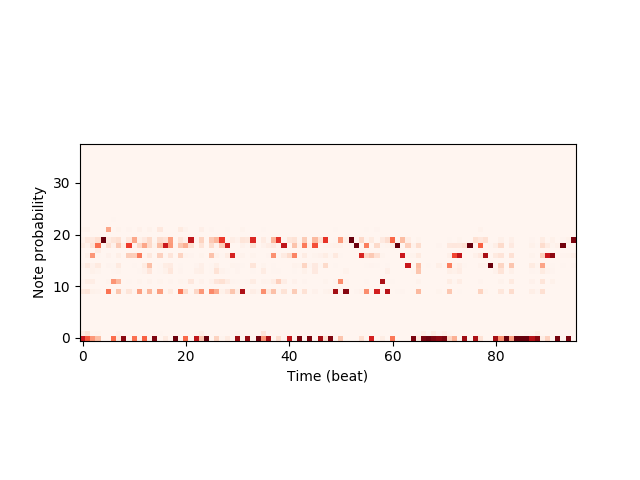

Supplement: Supplemental Information 3 [file peerj-cs-09-1356-s003.zip › Results/magenta+galician/new_rule_set/section_B/q/pre_rl64.png]

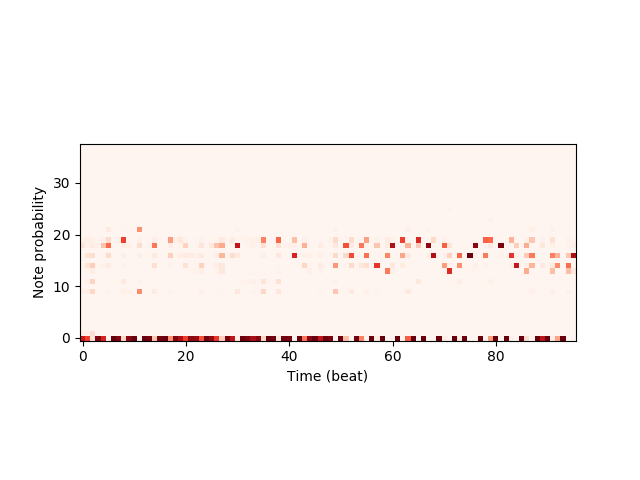

Supplement: Supplemental Information 3 [file peerj-cs-09-1356-s003.zip › Results/magenta+galician/new_rule_set/section_B/q/pre_rl70.png]

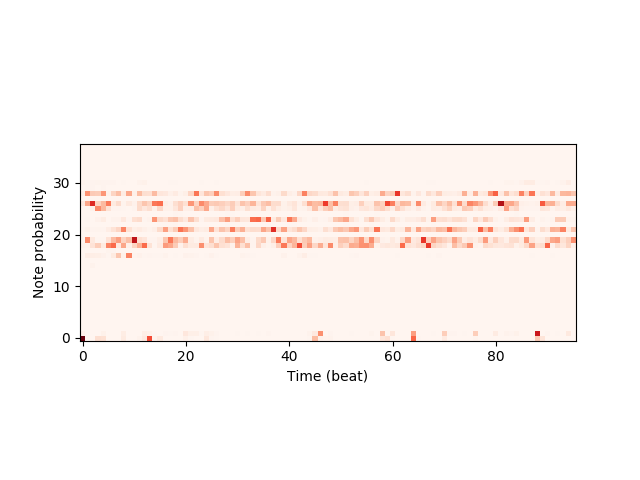

Supplement: Supplemental Information 3 [file peerj-cs-09-1356-s003.zip › Results/magenta+galician/new_rule_set/section_B/q/pre_rl76.png]

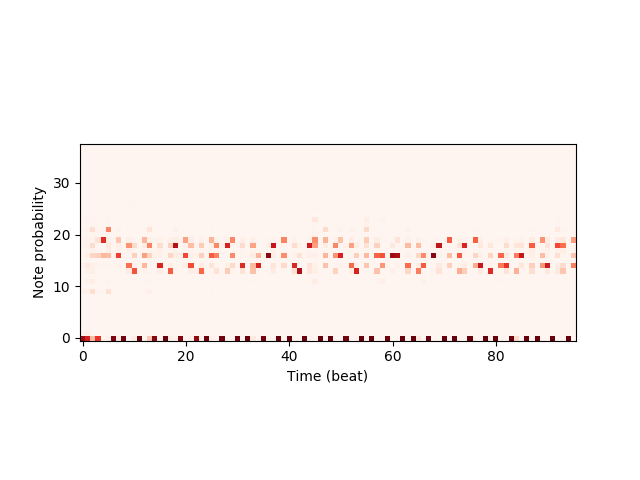

Supplement: Supplemental Information 3 [file peerj-cs-09-1356-s003.zip › Results/magenta+galician/new_rule_set/section_B/q/pre_rl77.png]

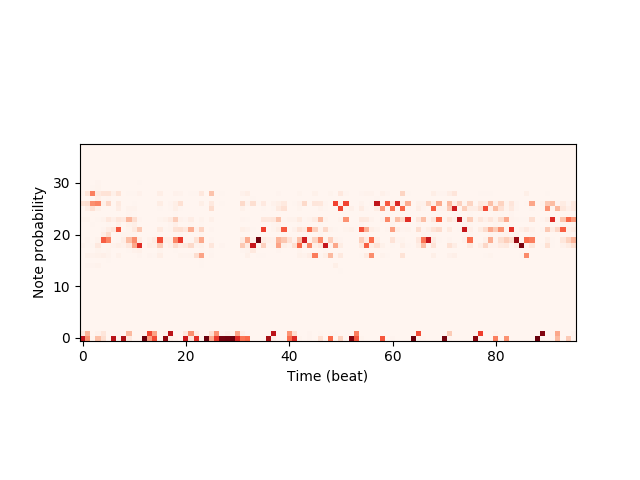

Supplement: Supplemental Information 3 [file peerj-cs-09-1356-s003.zip › Results/magenta+galician/new_rule_set/section_B/q/pre_rl78.png]

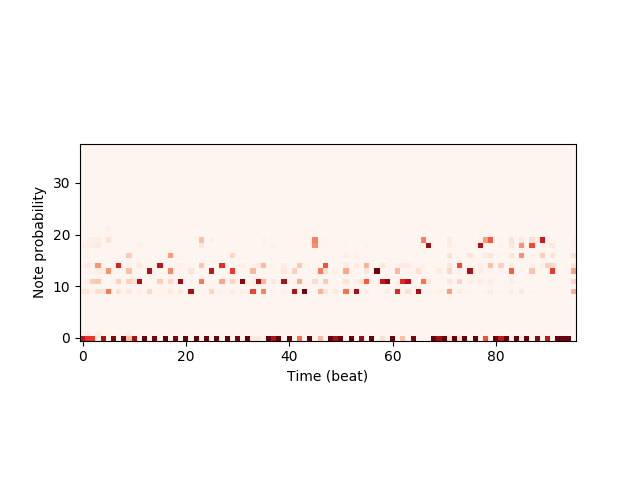

Supplement: Supplemental Information 3 [file peerj-cs-09-1356-s003.zip › Results/magenta+galician/new_rule_set/section_B/q/pre_rl79.png]

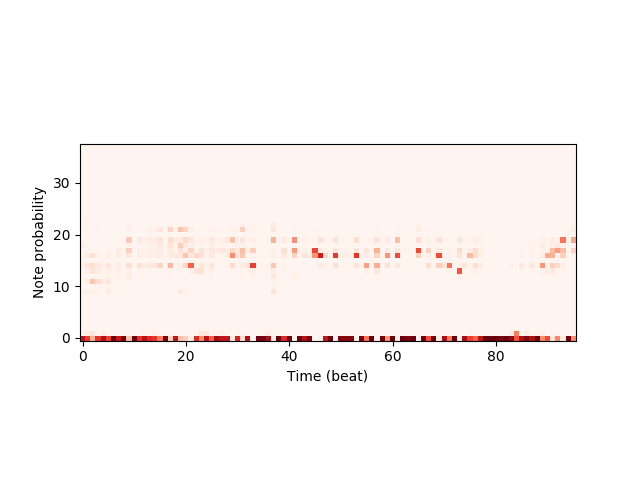

Supplement: Supplemental Information 3 [file peerj-cs-09-1356-s003.zip › Results/magenta+galician/new_rule_set/section_B/q/pre_rl96.png]

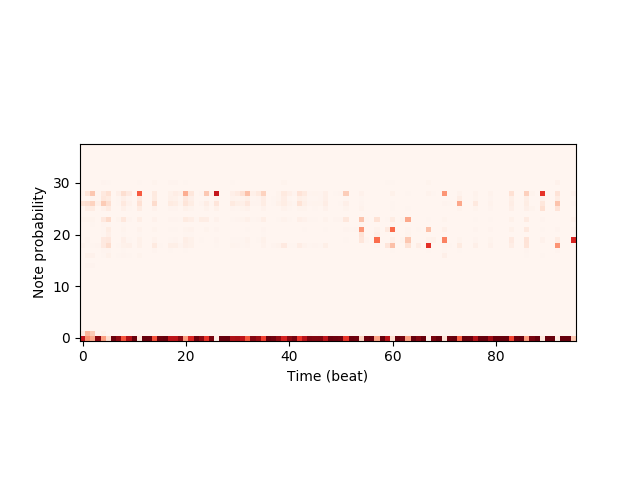

Supplement: Supplemental Information 3 [file peerj-cs-09-1356-s003.zip › Results/magenta+galician/new_rule_set/section_B/q/pre_rl60.png]

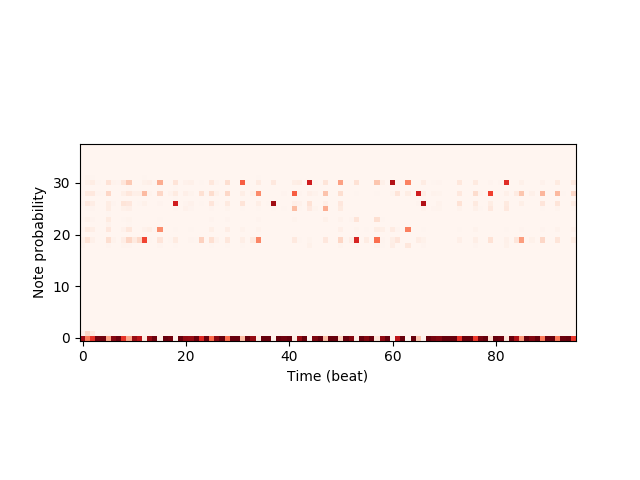

Supplement: Supplemental Information 3 [file peerj-cs-09-1356-s003.zip › Results/magenta+galician/new_rule_set/section_B/q/pre_rl61.png]

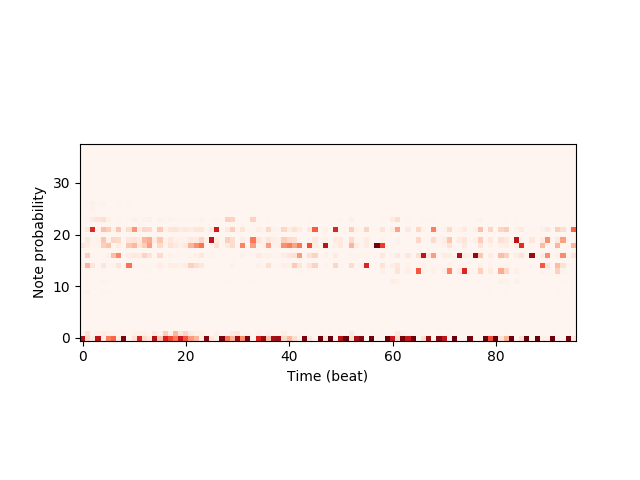

Supplement: Supplemental Information 3 [file peerj-cs-09-1356-s003.zip › Results/magenta+galician/new_rule_set/section_B/q/pre_rl65.png]

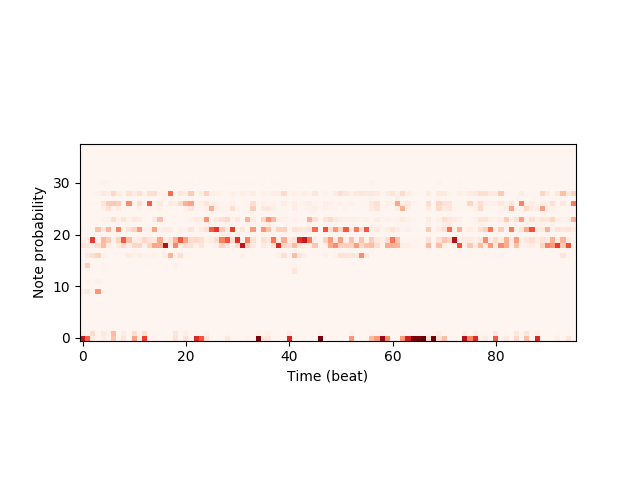

Supplement: Supplemental Information 3 [file peerj-cs-09-1356-s003.zip › Results/magenta+galician/new_rule_set/section_B/q/pre_rl88.png]

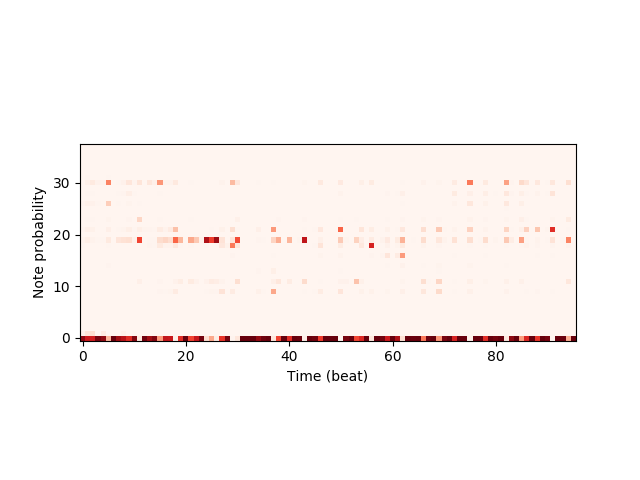

Supplement: Supplemental Information 3 [file peerj-cs-09-1356-s003.zip › Results/magenta+galician/new_rule_set/section_B/q/pre_rl16.png]

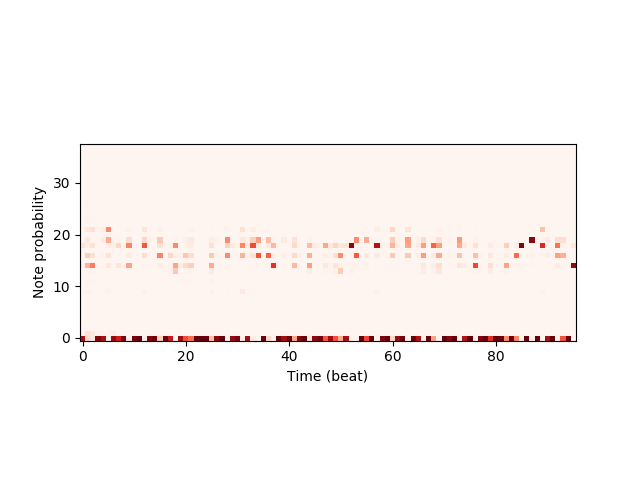

Supplement: Supplemental Information 3 [file peerj-cs-09-1356-s003.zip › Results/magenta+galician/new_rule_set/section_B/q/pre_rl39.png]

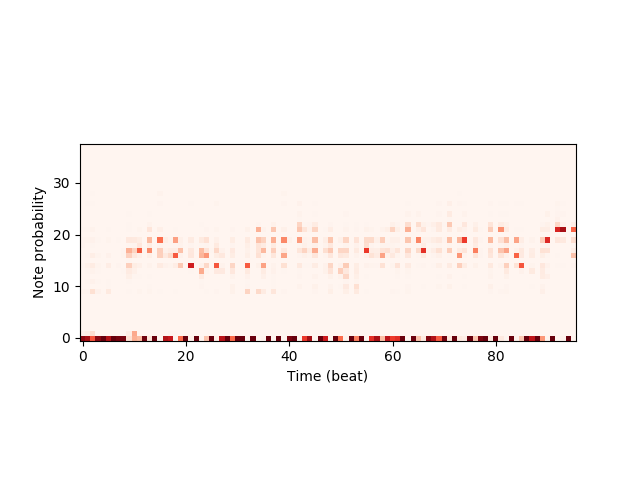

Supplement: Supplemental Information 3 [file peerj-cs-09-1356-s003.zip › Results/magenta+galician/new_rule_set/section_B/q/pre_rl53.png]

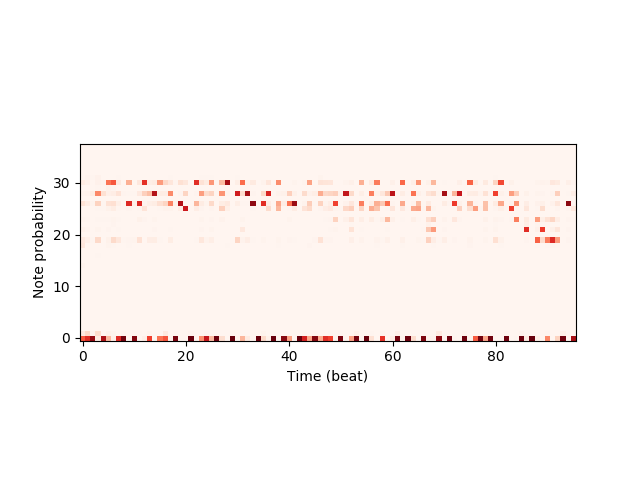

Supplement: Supplemental Information 3 [file peerj-cs-09-1356-s003.zip › Results/magenta+galician/new_rule_set/section_B/q/pre_rl62.png]

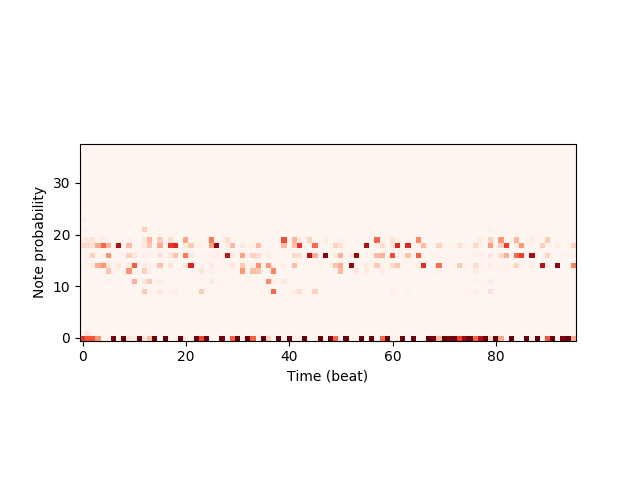

Supplement: Supplemental Information 3 [file peerj-cs-09-1356-s003.zip › Results/magenta+galician/new_rule_set/section_B/q/pre_rl66.png]

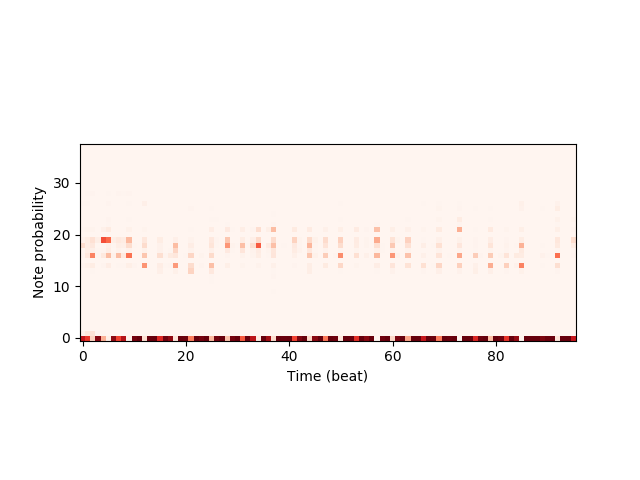

Supplement: Supplemental Information 3 [file peerj-cs-09-1356-s003.zip › Results/magenta+galician/new_rule_set/section_B/q/pre_rl81.png]

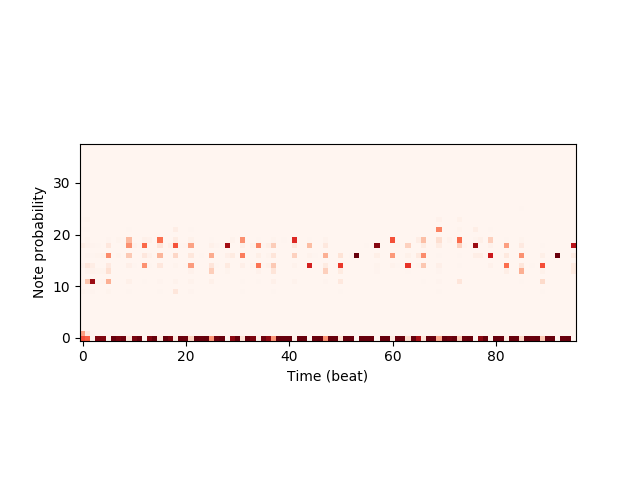

Supplement: Supplemental Information 3 [file peerj-cs-09-1356-s003.zip › Results/magenta+galician/new_rule_set/section_B/q/pre_rl83.png]

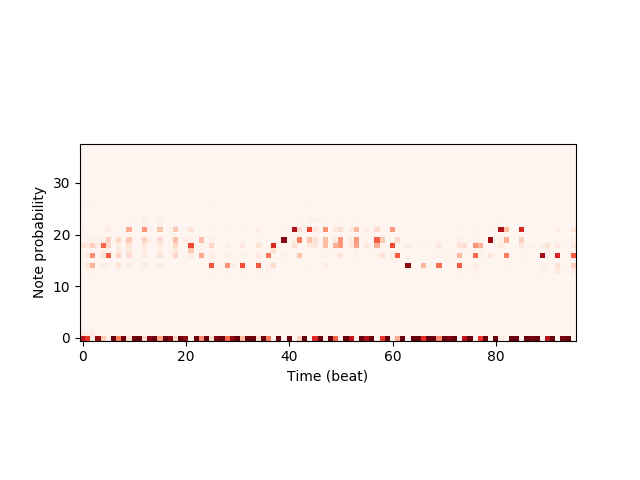

Supplement: Supplemental Information 3 [file peerj-cs-09-1356-s003.zip › Results/magenta+galician/new_rule_set/section_B/q/pre_rl9.png]

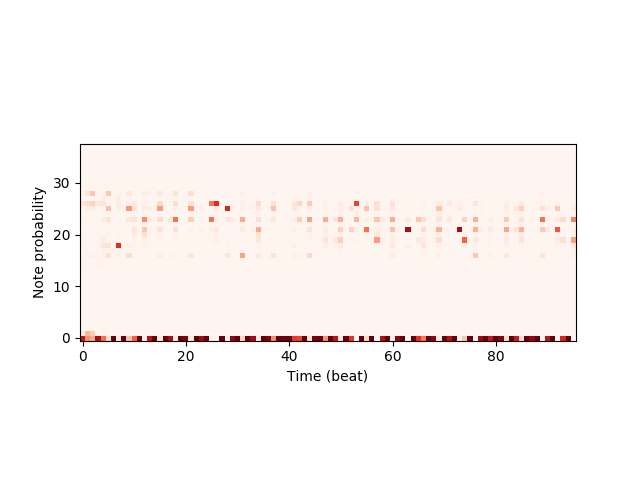

Supplement: Supplemental Information 3 [file peerj-cs-09-1356-s003.zip › Results/magenta+galician/new_rule_set/section_B/q/pre_rl90.png]

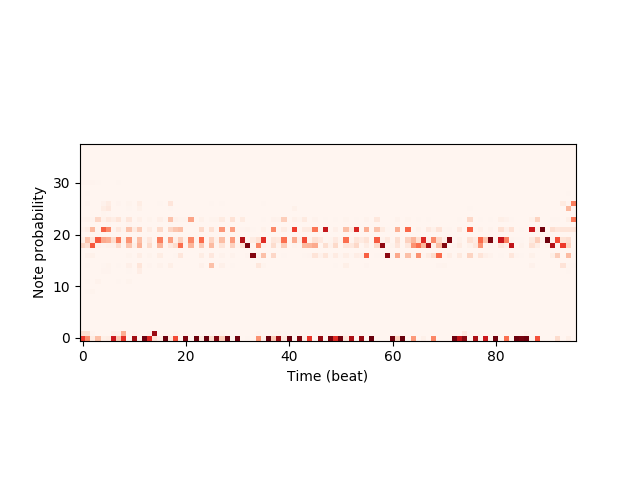

Supplement: Supplemental Information 3 [file peerj-cs-09-1356-s003.zip › Results/magenta+galician/new_rule_set/section_B/q/pre_rl0.png]

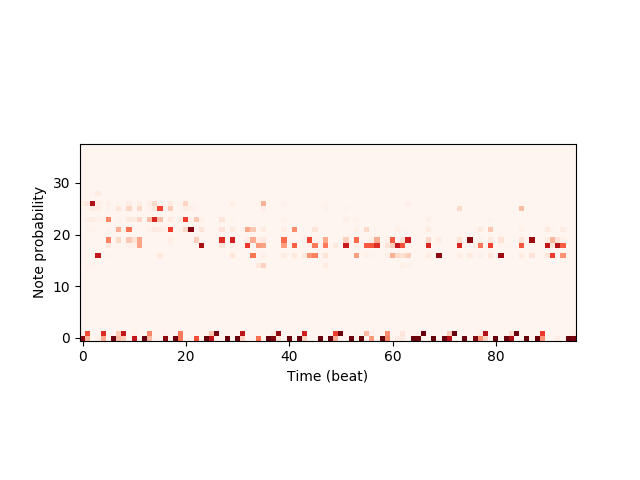

Supplement: Supplemental Information 3 [file peerj-cs-09-1356-s003.zip › Results/magenta+galician/new_rule_set/section_B/q/pre_rl49.png]

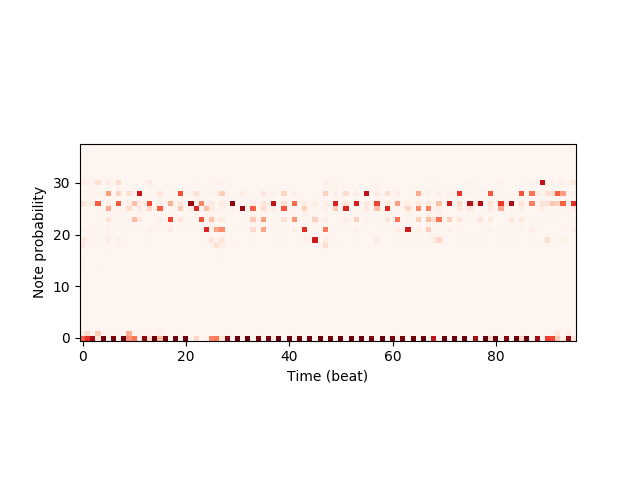

Supplement: Supplemental Information 3 [file peerj-cs-09-1356-s003.zip › Results/magenta+galician/new_rule_set/section_B/q/pre_rl5.png]

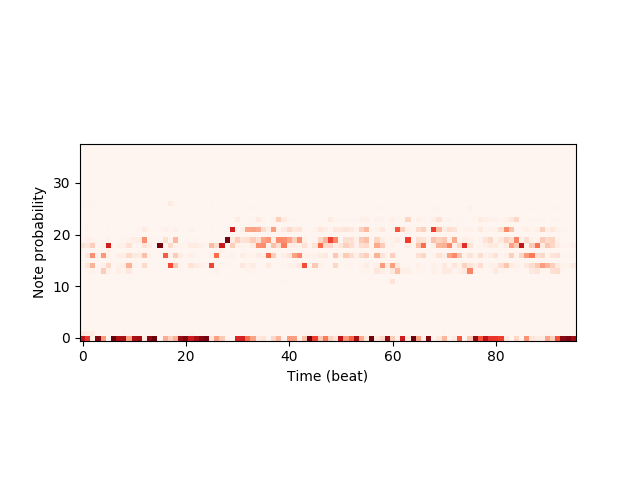

Supplement: Supplemental Information 3 [file peerj-cs-09-1356-s003.zip › Results/magenta+galician/new_rule_set/section_B/q/pre_rl6.png]

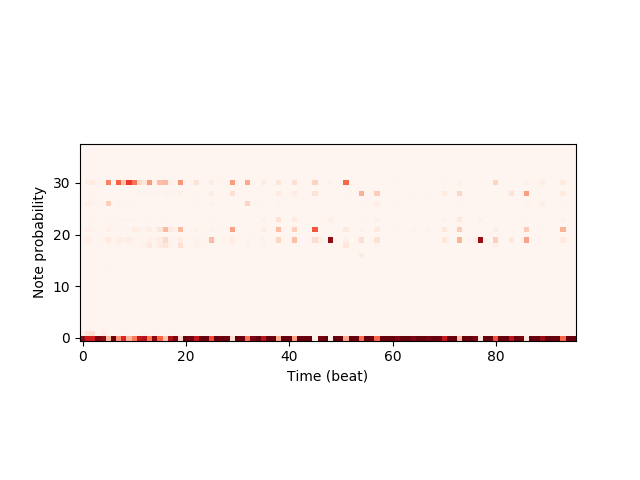

Supplement: Supplemental Information 3 [file peerj-cs-09-1356-s003.zip › Results/magenta+galician/new_rule_set/section_B/q/pre_rl93.png]

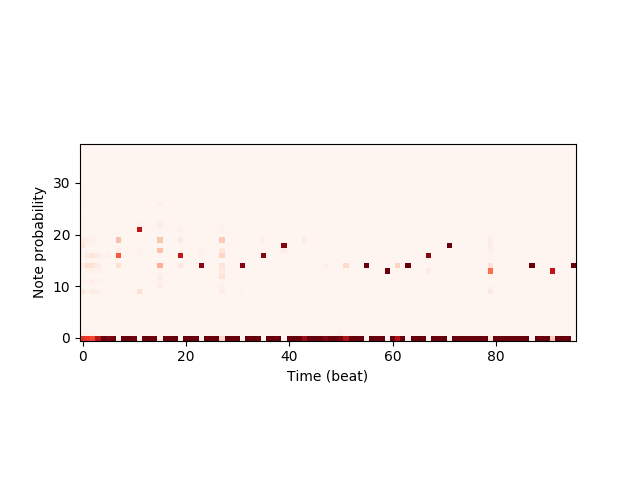

Supplement: Supplemental Information 3 [file peerj-cs-09-1356-s003.zip › Results/magenta+galician/new_rule_set/section_B/q/pre_rl98.png]

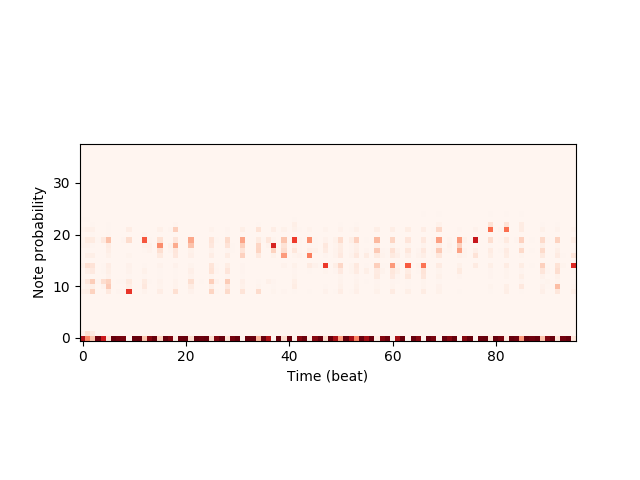

Supplement: Supplemental Information 3 [file peerj-cs-09-1356-s003.zip › Results/magenta+galician/new_rule_set/section_B/q/pre_rl14.png]

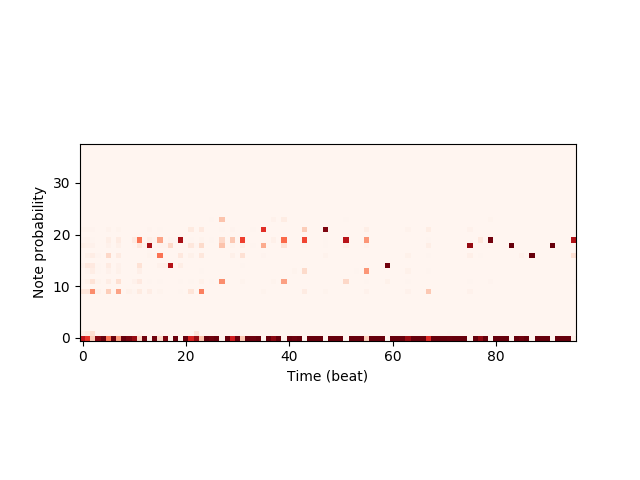

Supplement: Supplemental Information 3 [file peerj-cs-09-1356-s003.zip › Results/magenta+galician/new_rule_set/section_B/q/pre_rl17.png]

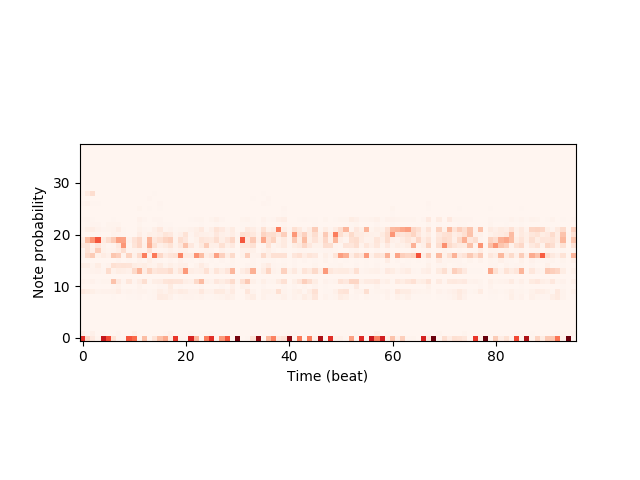

Supplement: Supplemental Information 3 [file peerj-cs-09-1356-s003.zip › Results/magenta+galician/new_rule_set/section_B/q/pre_rl21.png]

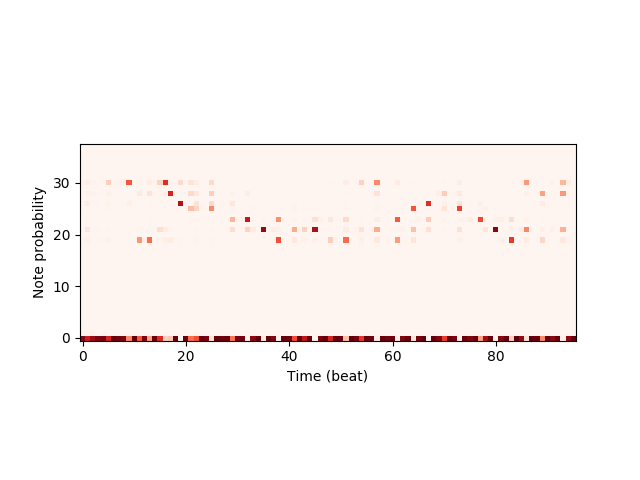

Supplement: Supplemental Information 3 [file peerj-cs-09-1356-s003.zip › Results/magenta+galician/new_rule_set/section_B/q/pre_rl55.png]

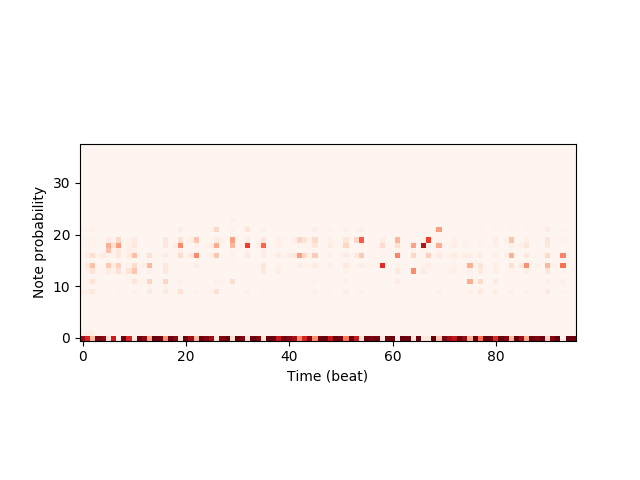

Supplement: Supplemental Information 3 [file peerj-cs-09-1356-s003.zip › Results/magenta+galician/new_rule_set/section_B/q/pre_rl67.png]

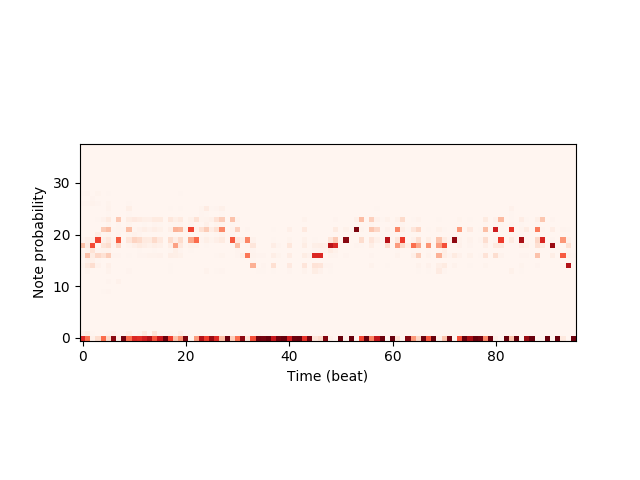

Supplement: Supplemental Information 3 [file peerj-cs-09-1356-s003.zip › Results/magenta+galician/new_rule_set/section_B/q/pre_rl75.png]

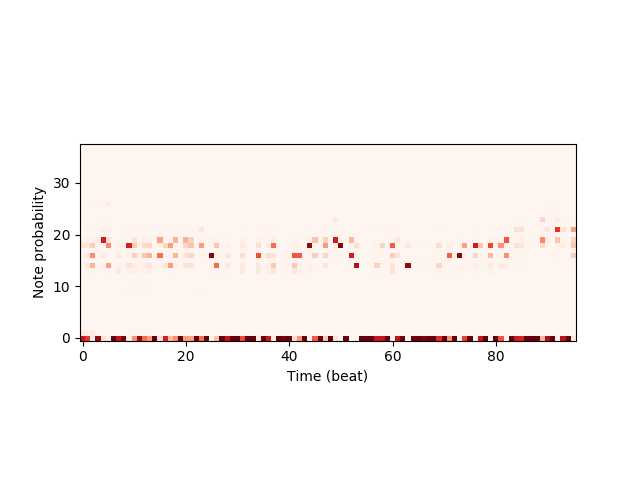

Supplement: Supplemental Information 3 [file peerj-cs-09-1356-s003.zip › Results/magenta+galician/new_rule_set/section_B/q/pre_rl86.png]

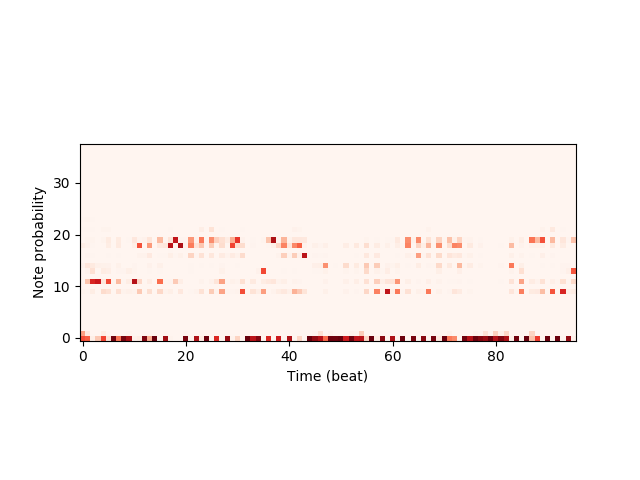

Supplement: Supplemental Information 3 [file peerj-cs-09-1356-s003.zip › Results/magenta+galician/new_rule_set/section_B/q/pre_rl18.png]

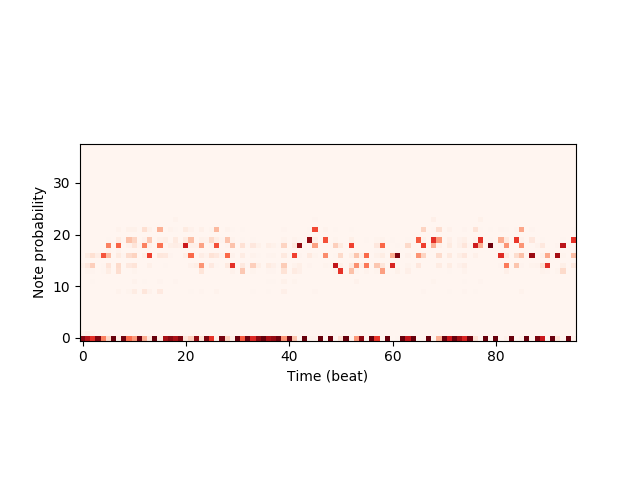

Supplement: Supplemental Information 3 [file peerj-cs-09-1356-s003.zip › Results/magenta+galician/new_rule_set/section_B/q/pre_rl19.png]

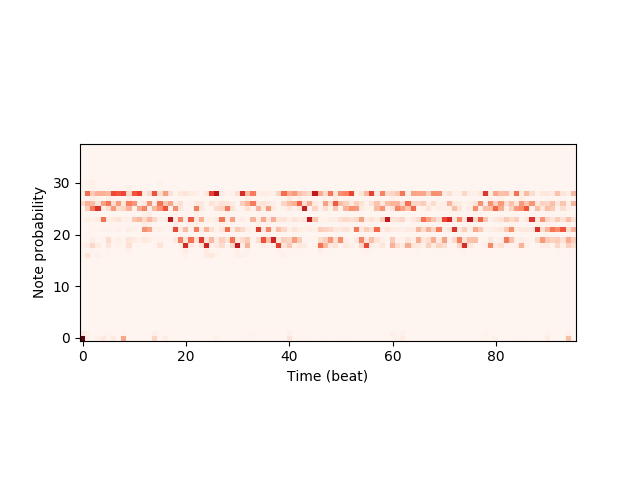

Supplement: Supplemental Information 3 [file peerj-cs-09-1356-s003.zip › Results/magenta+galician/new_rule_set/section_B/q/pre_rl23.png]

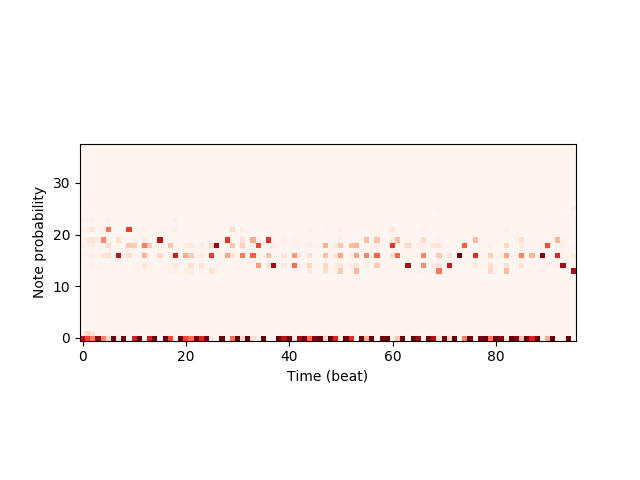

Supplement: Supplemental Information 3 [file peerj-cs-09-1356-s003.zip › Results/magenta+galician/new_rule_set/section_B/q/pre_rl24.png]

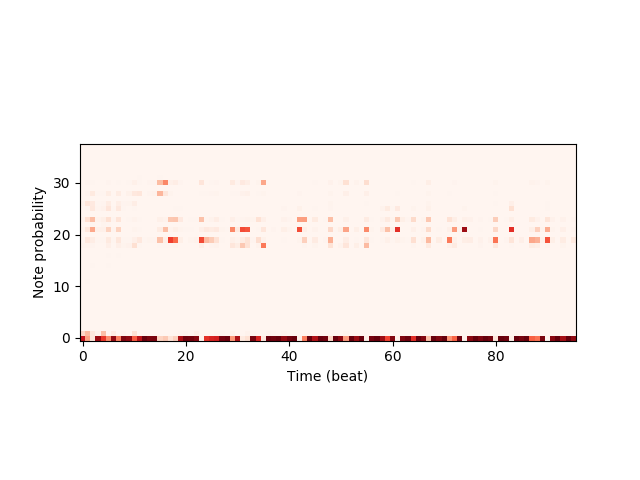

Supplement: Supplemental Information 3 [file peerj-cs-09-1356-s003.zip › Results/magenta+galician/new_rule_set/section_B/q/pre_rl47.png]

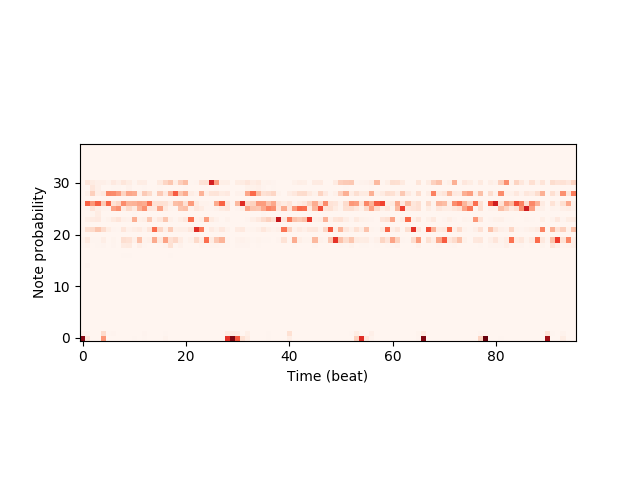

Supplement: Supplemental Information 3 [file peerj-cs-09-1356-s003.zip › Results/magenta+galician/new_rule_set/section_B/q/pre_rl50.png]

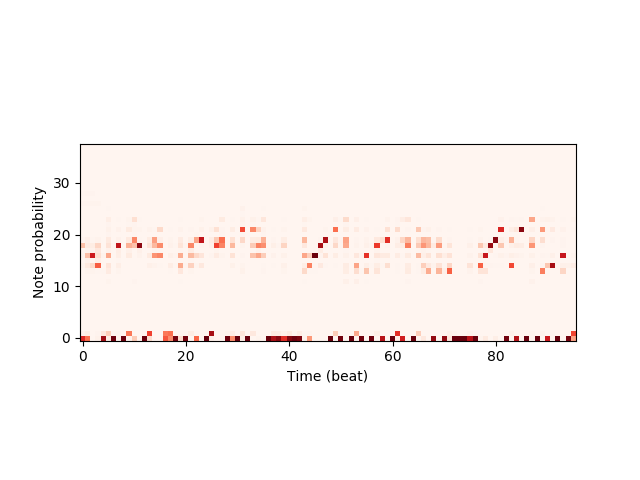

Supplement: Supplemental Information 3 [file peerj-cs-09-1356-s003.zip › Results/magenta+galician/new_rule_set/section_B/q/pre_rl52.png]

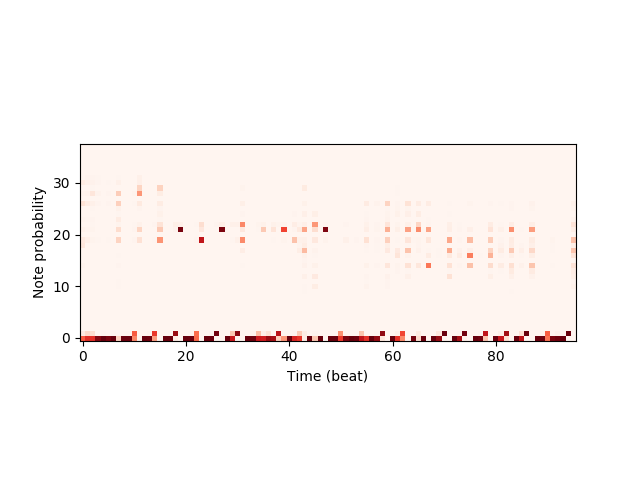

Supplement: Supplemental Information 3 [file peerj-cs-09-1356-s003.zip › Results/magenta+galician/new_rule_set/section_B/q/pre_rl59.png]

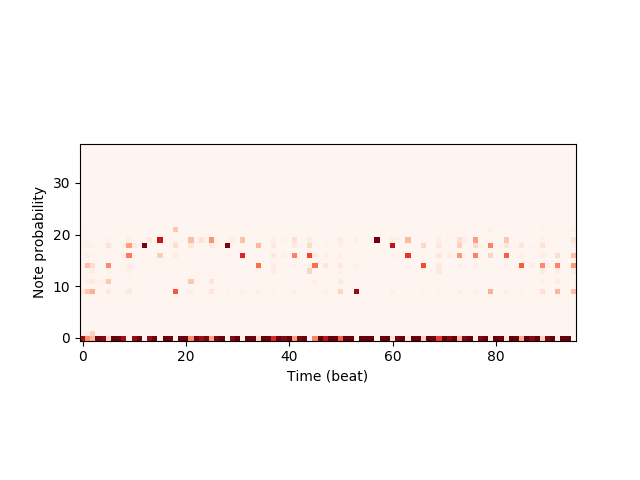

Supplement: Supplemental Information 3 [file peerj-cs-09-1356-s003.zip › Results/magenta+galician/new_rule_set/section_B/q/pre_rl74.png]

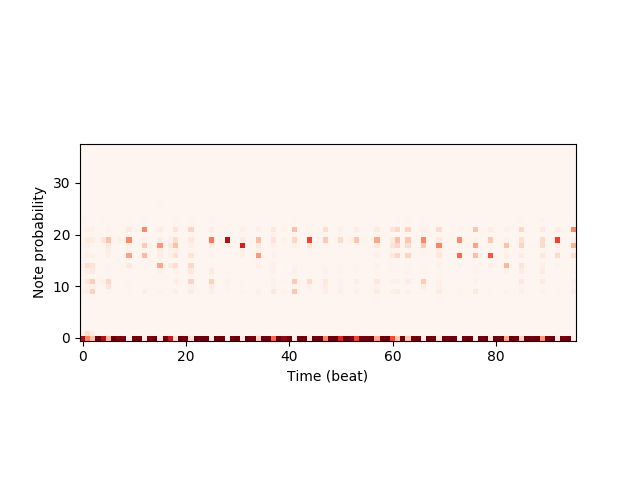

Supplement: Supplemental Information 3 [file peerj-cs-09-1356-s003.zip › Results/magenta+galician/new_rule_set/section_B/q/pre_rl95.png]

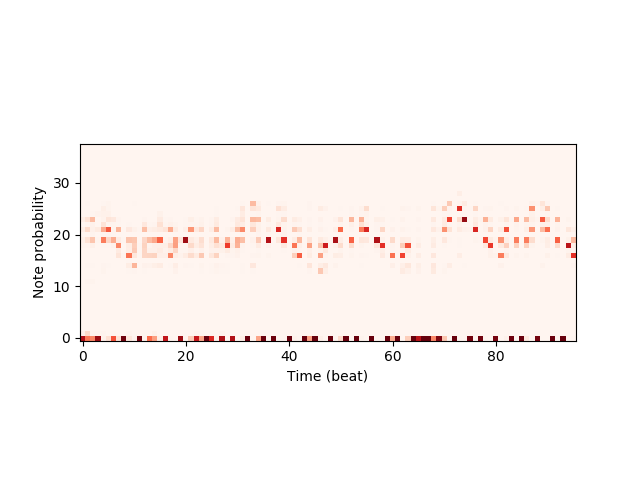

Supplement: Supplemental Information 3 [file peerj-cs-09-1356-s003.zip › Results/magenta+galician/new_rule_set/section_B/q/pre_rl1.png]

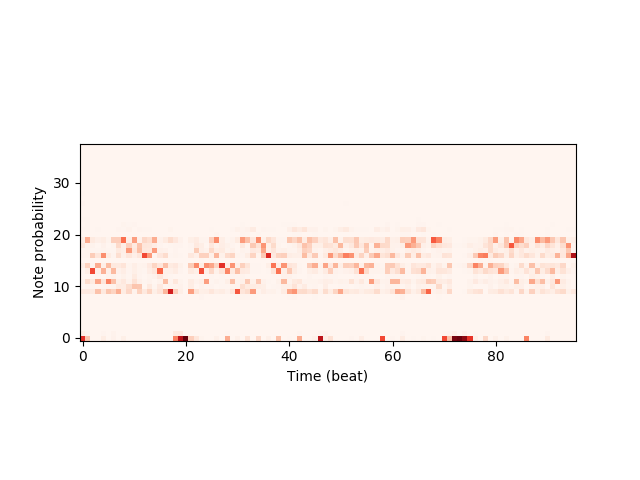

Supplement: Supplemental Information 3 [file peerj-cs-09-1356-s003.zip › Results/magenta+galician/new_rule_set/section_B/q/pre_rl13.png]

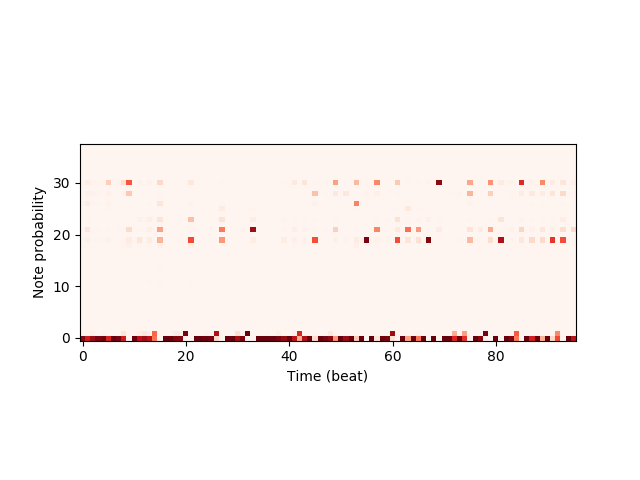

Supplement: Supplemental Information 3 [file peerj-cs-09-1356-s003.zip › Results/magenta+galician/new_rule_set/section_B/q/pre_rl40.png]

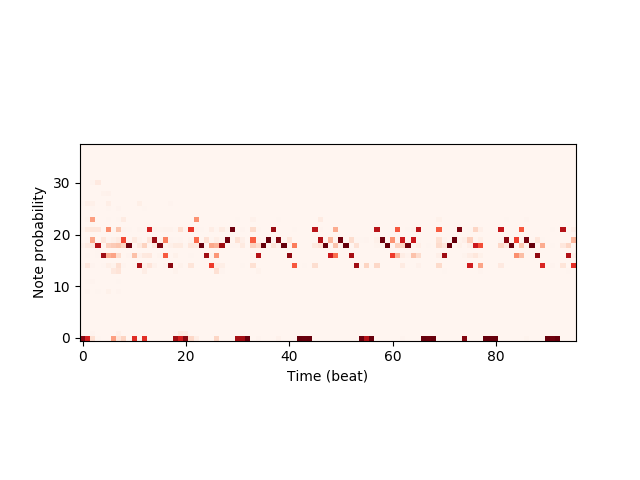

Supplement: Supplemental Information 3 [file peerj-cs-09-1356-s003.zip › Results/magenta+galician/new_rule_set/section_B/q/pre_rl91.png]

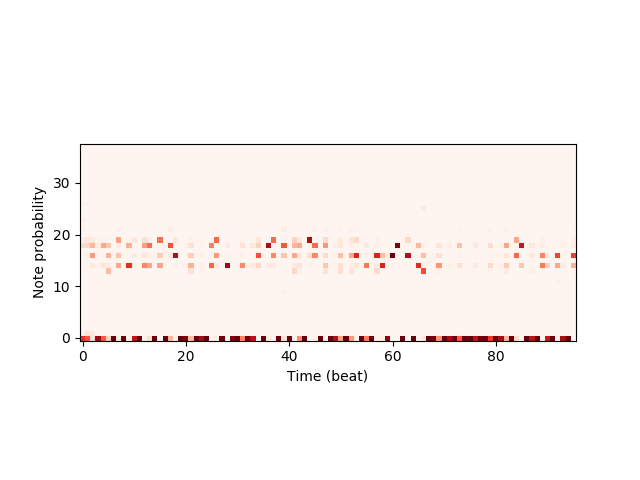

Supplement: Supplemental Information 3 [file peerj-cs-09-1356-s003.zip › Results/magenta+galician/new_rule_set/section_B/q/pre_rl92.png]

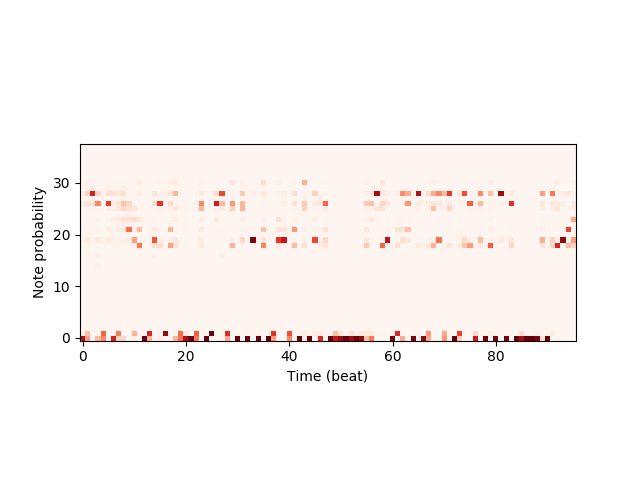

Supplement: Supplemental Information 3 [file peerj-cs-09-1356-s003.zip › Results/magenta+galician/new_rule_set/section_B/q/pre_rl26.png]
